# Two‐Step Tandem Catalysis for High‐Efficiency Ammonia Synthesis Via Nitrate Reduction on Anion‐Intercalated CoNi LDH and Cu/Cu_2_O

**DOI:** 10.1002/advs.202502262

**Published:** 2025-04-15

**Authors:** Changzheng Lin, Weijia Li, Hao Chen, Jiangtao Feng, Mengyuan Zhu, Jinwen Shi, Mingtao Li, Bo Hou, Zhenyu Wang, Xin Chen, Jia Liu, Wei Yan

**Affiliations:** ^1^ Department of Environmental Science & Engineering School of Energy and Power Engineering Xi'an Jiaotong University 28 Xianning West Road Xi'an 710049 China; ^2^ International Research Center for Renewable Energy (IRCRE) State Key Laboratory of Multiphase Flow in Power Engineering School of Energy and Power Engineering Xi'an Jiaotong University 28 Xianning West Road Xi'an 710049 China; ^3^ School of Physics and Astronomy Cardiff University, The Parade Cardiff Wales CF24 3AA United Kingdom; ^4^ Instrument Analysis Center of Xi'an Jiaotong University Xi'an Jiaotong University Xi'an 710049 China

**Keywords:** Ammonia synthesis, CuO nanowire, LDH layer spacing, Nitrate reduction reaction, Tandem catalytic kinetic

## Abstract

Ammonia is essential across industry, agriculture, and as a future carbon‐free energy carrier. Electrocatalytic nitrate reduction (NitRR) offers a sustainable path for removing nitrate contaminants from wastewater and groundwater while using abundant nitrate ions as nitrogen sources under eco‐friendly conditions. However, the NitRR pathway, which involves sequential reactions, poses challenges in synchronizing the rate of nitrate‐to‐nitrite conversion with the subsequent reduction of nitrite to ammonia, particularly as the initial reduction step is rate‐limiting. This study presents a CoNi layered double hydroxide (LDH) approach to finely control hydrogen radical (*H) supply, paired with Cu/Cu_2_O redox coupling, to achieve optimal rate matching. CoNi LDH is engineered with various anion intercalations (NO_3_^−^, Cl^−^, SO_4_^2−^, MoO_4_^2−^, WO_4_^2−^) to regulate *H capacity. By integrating Cu/Cu_2_O and CoNi LDH, tandem kinetic descriptors, including a volcano curve, are employed to predict rate constants, facilitating ideal kinetic matching for efficient ammonia synthesis. The optimized MoO_4_‐CoNi LDH/CuO NW/CF electrode demonstrated exceptional performance, achieving a 99.78% Faraday efficiency, a yield of 1.12 mmol cm^−2^ h^−1^ at −0.2 V vs. RHE, and robust 14‐h stability. The model descriptors effectively elucidated the kinetic pathway, linking reaction rates and factors impacting ammonia production.

## Graphical abstract

By integrating Cu/Cu_2_O and CoNi LDH, tandem kinetic descriptors, including a volcano curve, are employed to predict rate constants, facilitating ideal kinetic matching for efficient ammonia synthesis. The highest ammonia yield (1.12 mmol cm^−2^ h^−1^) and Faraday efficiency (99.78%) are achieved by rate matching between Cu/Cu_2_O and MoO_4_‐CoNi LDH.

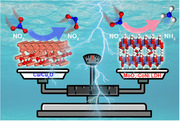

## 1 Introduction

Ammonia (NH_3_), the predominant chemical compound in industrial applications, serves as the primary feedstock for agricultural fertilizers and holds promising potential as a hydrogen‐rich fuel in the context of achieving carbon neutrality.^[^ ^1^, ^2^ ^]^ The conventional approach for ammonia synthesis (Haber‐Bosch method) requires high temperature and pressure conditions, and involves hydrogen production from fossil fuels and nitrogen preparation.^[^ ^3^ ^]^ Sustainable ammonia production has become a focal point and challenge in ammonia synthesis research.^[^ ^4^, ^5^, ^6^, ^7^ ^]^ Excessive fertilizer usage and discharge of industrial effluents have led to elevated levels of nitrates in surface and groundwater, posing a threat to human health.^[^ ^8^ ^]^ Therefore, electrocatalytic nitrate reduction to ammonia utilizing nitrate as a nitrogen source and water as a hydrogen source offers advantages such as environmental restoration and energy storage under milder conditions.^[^ ^9^, ^10^, ^11^, ^12^ ^]^

Significant advancements have been achieved in both fundamental research and practical applications of electrocatalytic nitrate reduction for ammonia.^[^ ^13^, ^14^ ^]^ Recently, several studies have reported the utilization of two‐step tandem reaction catalysts for efficient electrocatalytic reduction of nitrate to ammonia. The electrocatalytic process involves sequential reduction of nitrate to nitrite and subsequent conversion of nitrite to ammonia through direct electron transfer and hydrogen radical reduction.^[^ ^15^, ^16^, ^17^, ^18^ ^]^ Notably, the concentration variation of nitrite serves as a crucial indicator reflecting the disparity in kinetics between these two steps within the overall electrocatalytic process.^[^ ^19^, ^20^, ^21^, ^22^ ^]^ Despite numerous investigations dedicated to exploring kinetic matching in two‐step tandem reactions, how to quantitatively describe the rate kinetics remains pivotal for achieving effective kinetic matching.

In this study, a series of anionic intercalations CoNi LDH/CuO NW/CF were synthesized to achieve nitrate reduction for ammonia production. Encouragingly, MoO_4_‐CoNi LDH/CuO NW/CF exhibited high ammonia‐forming activity (1.12 mmol cm^−2^ h^−1^) and Faraday efficiency (FE) (99.78%) at an ultra‐low potential of −0.2 V vs. RHE, surpassing conventional Cu catalysts. By monitoring the concentrations of nitrate, nitrite and ammonia in the NitRR process, a two‐step tandem reaction kinetic process was constructed, and the performance relationship between the two‐step tandem reaction kinetic coefficient and ammonia production was elucidated by a two‐step tandem reaction kinetic model descriptor and volcano curve description. The kinetic‐descriptors approach is based on the analysis of the relationship between the rate constants for the sequential reactions of nitrate reduction to nitrite (*k*_1_) and nitrite reduction to ammonia (*k*_2_). Additionally, volcanic curve‐descriptors based on energy barriers for *H and H_2_ release were established. The kinetic‐descriptor and volcano curve‐descriptor were successfully applied to elucidate the two‐step tandem kinetic reaction pathway, clarify the relationship between *k*_1_ and *k*_2_, identify influencing factors, and assess the impact on ammonia production performance. The results from in situ electrochemical measurements and density functional theory (DFT) calculations elucidate the distinct roles played by Cu_2_O (CuO reduction product) and CoNi LDH in the sequential reduction steps from NO_3_^−^ to NO_2_^−^ and then from NO_2_^−^ to NH_3_, respectively. As‐proposed tandem reaction kinetics model and descriptors offer a promising tool for quantitatively describing NitRR processes and optimizing reaction conditions.

## 2 Results and Discussion

### 2.1 Catalyst Design and Characterization

**Figure 1:**
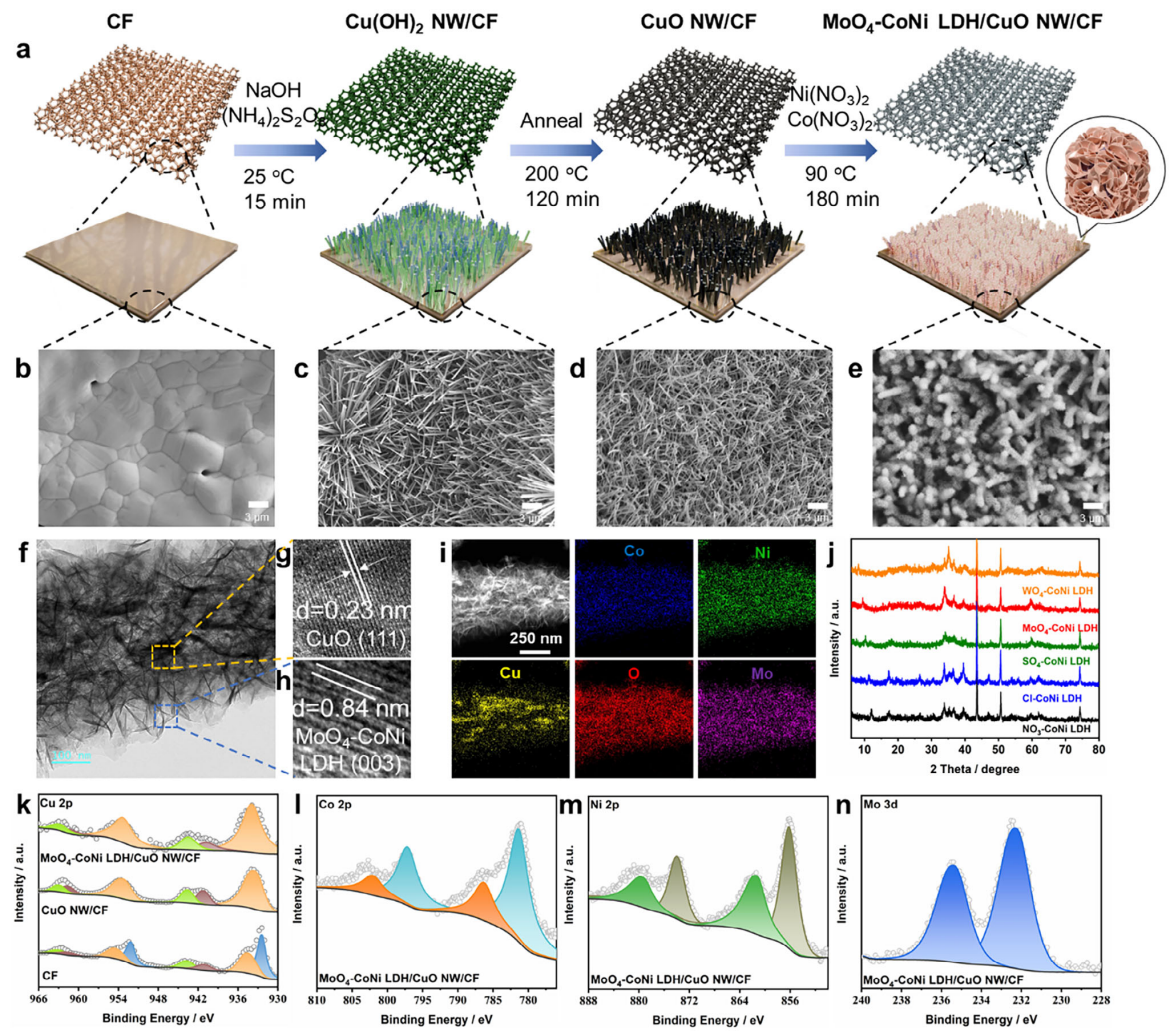
Structural characterizations of catalysts. a) Schematic illustration of the preparation of a MoO_4_‐CoNi LDH/CuO NW/CF binary cooperative catalyst. SEM images of the CF b), Cu(OH)_2_ NW/CF c), CuO NW/CF d) and MoO_4_‐CoNi LDH /CuO NW/CF e). f) TEM and g,h) high‐resolution TEM (HRTEM) images of MoO_4_‐CoNi LDH/CuO NW/CF. i) TEM element mapping images of MoO_4_‐CoNi LDH/CuO NW. j) XRD patterns of NO_3_‐NiCo LDH/CuO NW/CF, Cl‐NiCo LDH/CuO NW/CF, SO_4_‐NiCo LDH/CuO NW/CF, MoO_4_‐NiCo LDH/CuO NW/CF and WO_4_‐NiCo LDH/CuO NW/CF. k) Cu 2p, l) Co 2p, m) Ni 2p, and n) Mo 3d XPS spectra of MoO_4_‐CoNi LDH/CuO NW/CF.

The synthesis process of the MoO_4_‐CoNi LDH/CuO NW/CF catalyst is illustrated in **Figure** **1**a. Cu(OH)_2_ NW/CF was prepared through chemical oxidation on the copper foam (CF) matrix, and CuO NW/CF was obtained via calcination. MoO_4_‐CoNi LDH nanosheets was then grown on the surface of the CuO NW/CF composite substrate using a hydrothermal method, resulting in the formation of MoO_4_‐CoNi LDH/CuO NW/CF. In the preparation process, acid group anion‐NiCo LDH/CuO NW/CF was synthesized via a one‐pot hydrothermal method using different salts as precursors containing target anions. For NO_3_‐NiCo LDH/CuO NW/CF, Cl‐NiCo LDH/CuO NW/CF, and SO_4_‐NiCo LDH/CuO NW/CF, nickel and cobalt nitrates, chlorides, and sulfates were directly employed as reactants. In the synthesis of MoO_4_‐NiCo LDH/CuO NW/CF and WO_4_‐NiCo LDH/CuO NW/CF, nickel and cobalt nitrates served as sources for Ni^2+^ and Co^2+^, while sodium molybdate and sodium tungstate acted as sources for MoO_4_^2−^ and WO_4_^2−^. Urea decomposes under hydrothermal conditions, releasing NH_3_ and CO_2_. The hydrolysis of NH_3_ gradually increases the pH value of the solution. The slow release of OH^−^ creates a mild alkaline environment, allowing the hydrolysis of Co^2+^ and Ni^2+^ ions to be controlled. Although urea can gradually generate CO_2_ to form carbonate during the hydrothermal process, carbonate accumulation is a gradual phenomenon that does not affect intercalation unless longer reaction times are utilized.^[^ ^23^ ^]^

The morphology transition from CF to MoO_4_‐CoNi LDH/CuO NW/CF was observed by the scanning electron microscopy (SEM) images. In contrast to the smooth bare CF surface (Figure 1b), the Cu(OH)_2_ NW/CF electrode exhibited a dense and uniformly distributed arrangement of Cu(OH)_2_ nanowires (NW) (Figure 1c). After calcination, the Cu(OH)_2_ NW/CF transformed into disordered CuO NW/CF with a rough nanowire morphology (Figure 1d). The SEM images in Figure 1e demonstrated a typical composite of MoO_4_‐CoNi LDH/CuO NW/CF, where the vertical orientation of CuO NW was supported by the CF substrate while MoO_4_‐CoNi LDH nanosheets grew vertically on its surface features, with the nanowires exhibiting a length of ≈7.5 µm (Figure S1, Supporting Information). Elemental mapping analysis showed uniform distribution of Cu and O elements in partially oxidized CF surfaces, Cu(OH)_2_ NW/CF, and CuO NW/CF (Figures S2–S4, Supporting Information). The uniform distribution of Ni, Co, Mo, Cu, and O elements was observed in both the planar and vertical directions within the MoO_4_‐CoNi LDH/CuO NW/CF composite (Figures S5 and S6, Supporting Information). Meanwhile, we successfully synthesized CoNi LDH/CuO NW/CF electrodes with various anion intercalations and consistently observed the presence of a layered structure (Figure S7, Supporting Information). The EDS plot revealed a uniform distribution of Ni, Co, and X (X = Mo, W, S, N, Cl) within the electrodes (Figures S5 and S8–S11, Supporting Information). Table S1 (Supporting Information) presents the results of EDS elemental analysis indicating their respective contents. The ratio of Ni to Co in all electrodes was approximately 1:1, consistent with the feed ratio. Furthermore, the anion content constituted 4%‐10% of the total content for these three elements.

To obtain more comprehensive structural information, transmission electron microscopy (TEM) analysis was conducted on extracted MoO_4_‐CoNi LDH from MoO_4_‐CoNi LDH/CuO NW/CF. The TEM image in Figure S12 (Supporting Information) revealed MoO_4_‐CoNi LDH growth on CuO NWs (250 nm), resulting in a rough surface with an approximate diameter of 500 nm. The HRTEM image reveals that the *d*‐spacing values of 0.22 and 0.23 nm correspond to the (0 0 3) plane of MoO_4_‐CoNi LDH and the (1 1 1) plane of CuO, respectively (Figure 1f–h).^[^ ^24^, ^25^ ^]^ To further analyze the element composition and distribution of MoO_4_‐CoNi LDH/CuO NW, high‐angle annular dark‐field scanning transmission electron microscopy (HAADF‐STEM) and corresponding element mappings were acquired. As illustrated in Figure 1i, the distribution of Cu in the MoO_4_‐CoNi LDH/CuO NW is primarily observed in the core, while the elements O, Co, Ni, and Mo are evenly distributed throughout the MoO_4_‐CoNi LDH/CuO NW. The layer spacing (*d_interlayer_*) of the different electrodes was characterized by XRD analysis, as shown in Figure 1j. Located at 33.9°, 39.3°, 46.8°, 59.7°, and 61.8° diffraction peaks can be designated as representative of the nickel hydroxide hydrate sample's hydrotalcite structure (Ni(OH)_2_·0.75H_2_O), corresponding to crystal faces (1 0 1), (0 1 5), and (1 1 3). Additionally, the shift in diffraction peak positions for planes (0 0 3) and (0 0 6) reflects changes in the middle tier of the crystal lattice. The observed peaks at approximately 12°, 11.9°, 10.3°, 9.2°, and 8.2° for NO_3_‐NiCo LDH, Cl‐NiCo LDH, SO_4_‐NiCo LDH, MoO_4_‐NiCo LDH and WO_4_‐NiCo LDH respectively indicate that only anion insertion leads to enlargement of the intermediate layer without significant alteration to the crystal structure of NiCo LDH.^[^ ^23^ ^]^ According to Bragg's equation (Equation 1), the double interlayer spacings for NO_3_‐NiCo LDH, Cl‐NiCo LDH, SO_4_‐NiCo LDH, MoO_4_‐NiCo LDH and WO_4_‐NiCo LDH are determined as 0.73, 0.74, 0.86, 0.96, and 1.07 nm, respectively. This evolution is consistent with the size order of these anions, since NO_3_^−^ has a planar shape and its longitudinal size is even smaller than Cl^−^. The XRD results demonstrate the successful preparation of gradient double interlayers in NiCo LDH.

(1) 2dsinθ=nλ

In this equation, d represents the interplanar spacing, θ represents the angle between the incident X‐ray and the corresponding crystal plane, *λ* represents the wavelength of the X‐ray, and n represents the diffraction order.

The XPS measurements were conducted to determine the chemical states and elemental information of different electrodes. As shown in Figure 1k, the high‐resolution X‐ray photoelectron spectroscopic (XPS) spectrum of Cu 2p_3/2_ presents peaks at 932.5 and 934.7 eV (and the Cu 2p_1/2_ presents peaks at 954.6 and 952.2 eV), indicating the CF containes both zero‐valent Cu and oxidation generated Cu^2+^, which corresponds to metal Cu and CuO. The Cu 2p spectra of CuO NW/CF and MoO_4_‐CoNi LDH/CuO NW/CF exhibited prominent peaks at ≈934 and ≈954 eV for the Cu 2p_3/2_ and Cu 2p_1/2_ states, respectively, providing evidence that the three electrodes predominantly consist of Cu^2+^. The peaks of Cu 2p_3/2_ in MoO_4_‐CoNi LDH/CuO NW/CF slightly shift to higher binding energy compared with that in CuO NW/CF. Such corresponding shifts demonstrate that electron transfers from Cu to Ni and Mo occur in MoO_4_‐CoNi LDH/CuO hetero‐phase interface, which is mainly attributed that Ni (Electronegativity: 1.91 Pauling Scale) and Mo (Electronegativity: 2.16 Pauling Scale) is more electronegative than Cu (Electronegativity: 1.90 Pauling Scale).^[^ ^26^ ^]^ In the high‐resolution Co 2p spectrum (Figure 1l), the peaks at 799.2 and 783.3 eV corresponded to Co 2p_1/2_ and Co 2p_3/2_ signals of Co^2+^, while the peaks at 797.0 and 781.0 eV corresponded to Co 2p_1/2_ and Co 2p_3/2_ signals of Co^3+^ species.^[^ ^27^ ^]^ In the high‐resolution Ni 2p spectrum (Figure 1m), the peaks observed at energies of 873.8 and 856.0 eV were attributed to Ni 2p_1/2_ and Ni  2p_3/2_, respectively, whereas other peaks represented shake‐up satellite features.^[^ ^24^ ^]^ In the high‐resolution Mo 3 d spectrum (Figure 1n), peak positions at energies of approximately 235 and 232 eV were assigned to Mo^6+^ species.^[^ ^23^ ^]^ Figure S13 (Supporting Information) displays XPS patterns for other electrodes, revealing that both Co^2+^ and Co^3+^ are present in all the electrodes, while nickel is only observed as Ni^2+^. The elemental composition of the different anions can be clearly identified from the spectra. Based on the above findings, it can be concluded that NiCo LDH with various intercalated anion have been successfully synthesized.

### 2.2 Electrocatalytic NitRR Performance

**Figure 2:**
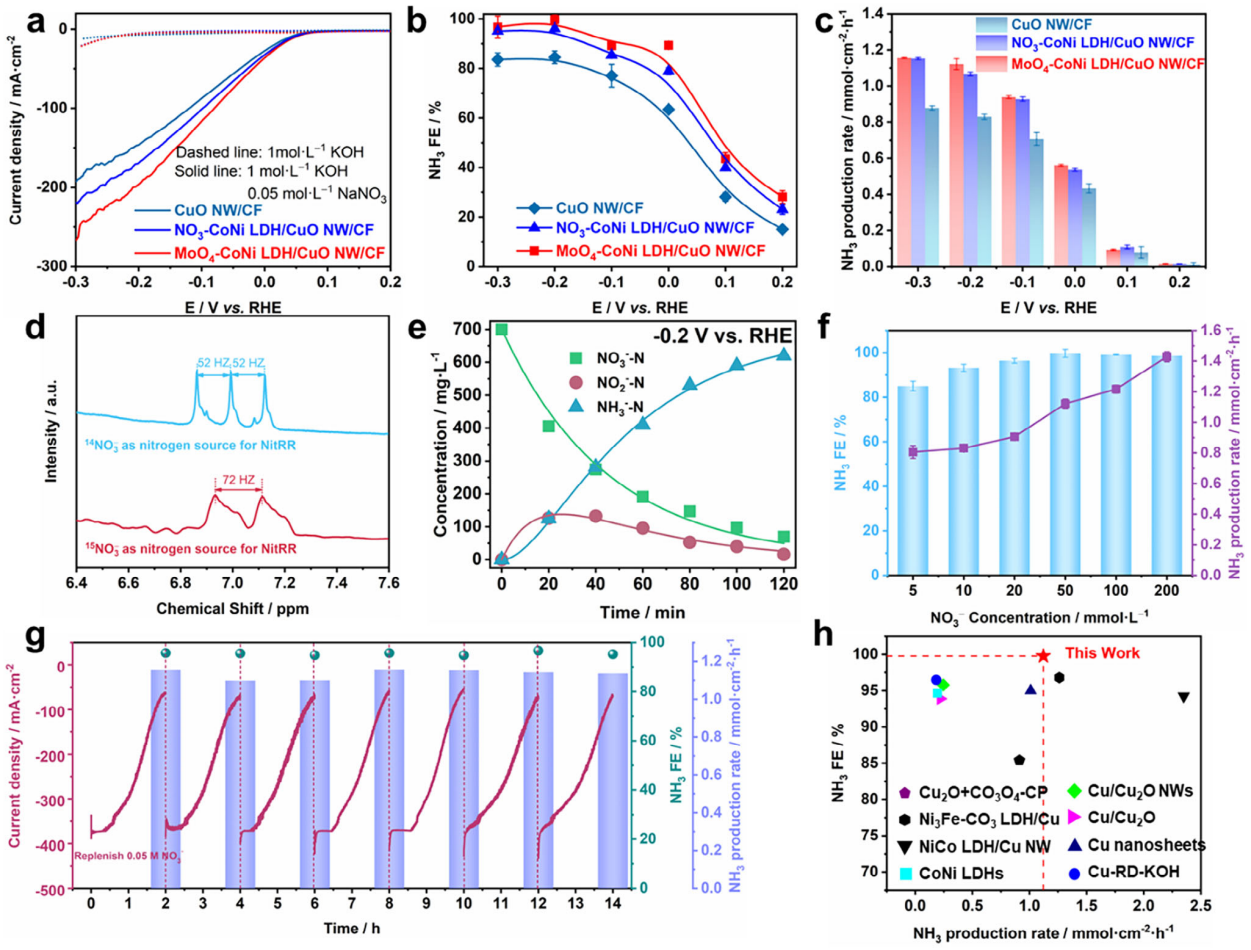
Electrochemical performance of catalysts. a) LSV curves depicting the performance of CuO NW/CF, NO_3_‐CoNi LDH/CuO NW/CF and MoO_4_‐CoNi LDH/CuO NW/CF in 1 mol L^−1^ KOH solution with (solid lines) or without (dashed lines) nitrate. The corresponding FE for NH_3_ b), NH_3_ production rate c) for CuO NW/CF, NO_3_‐CoNi LDH/CuO NW/CF and MoO_4_‐CoNi LDH/CuO NW/CF in a 1 mol L^−1^ KOH with 0.05 mol L^−1^ NO_3_^−^ electrolyte at different potentials. d) ^1^H NMR spectra of liquid products obtained through NitRR of ^14^N nitrate and ^15^N nitrate. e) Complete nitrate removal using MoO_4_‐CoNi LDH/CuO NW/CF with an initial 1 mol L^−1^ KOH containing ≈0.05 mol L^−1^ NO_3_^−^ electrolyte at −0.2 V vs. RHE. f) The NH_3_ FE and production rate on the heterogeneous MoO_4_‐CoNi LDH/CuO NW/CF catalyst at −0.2 V vs. RHE in the range of 0.005‐0.2 mol L^−1^ NO_3_^−^ at 1 mol L^−1^ KOH. g) Long‐term current‐time stability test of MoO_4_‐CoNi LDH /CuO NW/CF at −0.2 V vs. RHE in 1 mol L^−1^ KOH with 0.05 mol L^−1^ NO_3_^−^, employing a continuous flow system in an H‐type cell. h) Comparative analysis of electrochemical performance with benchmark reported catalysts (See detailed information in Table S3, Supporting Information).

The electrochemical NitRR performance of the CoNi LDH/CuO NW/CF was investigated under ambient temperature and pressure in a standard three‐electrode H‐type cell. In this study, NO_3_^−^, NO_2_^−^, and NH_3_ were quantified by UV‐vis spectrophotometry with calibration curves (Figures S14–S16, Supporting Information) and the data are shown in Table S2 (Supporting Information). An industrial wastewater with a relevant nitrate concentration of 0.05 mol L^−1^ was used in our electrolyte for the standard electrochemical characterizations of the catalysts. As shown in **Figure** **2**a, CuO NW/CF, NO_3_‐CoNi LDH/CuO NW/CF, and MoO_4_‐CoNi LDH/CuO NW/CF exhibit distinct LSV curves, with MoO_4_‐CoNi LDH/CuO NW/CF demonstrating exceptional intrinsic activity for nitrate reduction by achieving the highest reduction current density. NO_3_‐CoNi LDH/CuO NW/CF shows the second‐highest NitRR response current, while CuO NW/CF displays the poorest performance. Notably, at a reduction current of 250 mA cm^−2^, the potential of MoO_4_‐CoNi LDH/CuO NW/CF is merely −0.29 V vs. RHE. Furthermore, we investigated the NitRR performance of three catalysts at −0.2 V vs. RHE under different conditions(Figure S17, Supporting Information), and finally selected these three catalysts to study the catalytic products at different potentials to examine the NH_3_ FE (NH_3_ selectivity) of NitRR. As the potential decreases from 0.2 to −0.3 V vs. RHE, there is an initial increase followed by a decrease in NH_3_ FE, while the NO_2_^−^ by‐product exhibits a decreasing trend. At an applied potential of −0.2 V vs. RHE, MoO_4_‐CoNi LDH/CuO NW/CF demonstrated an NH_3_ FE sum close to 100%, whereas CuO NW/CF and NO_3_‐CoNi LDH/CuO NW/CF exhibited slightly lower values than 100%. This observation could be attributed to HER competition or the formation of unmonitored by‐products such as N_2_, NO_x_, and N_2_H_4_. Notably, CuO NW/CF displayed the highest FE value (84.51%) for NH_3_ production, while MoO_4_‐CoNi LDH/CuO NW/CF exhibited superior performance in hydrogenating NO_2_^−^ with its highest FE value reaching 99.78% for NH_3_ production. These findings suggest that CuO has limited ability to adsorb and reduce NO_2_^−^ into NH_3_ compared to MoO_4_‐CoNi LDH, which excels in this process.

The maximum NH_3_ FE achieved by MoO_4_‐CoNi LDH/CuO NW/CF was 99.78% at −0.2 V vs. RHE resulting in an NH_3_ yield of 1.12 mmol h^−1^ cm^−2^. Further negative shifting of the electric potential to −0.3 V vs. RHE led to increased NH_3_ yield (1.15 mmol h^−1^ cm^−2^) but decreased NH_3_ FE, which still reached a promising level of 96.76%. Although CuO NW/CF demonstrates excellent catalytic activity toward nitrate reduction into nitrite (NO_2_^−^), its limited protonation performance for converting nitrite into ammonia hindered efficient completion of tandem reaction processes involving nitrate deoxygenation and hydrogenation, thereby leading to poor overall efficiency or low NH_3_ yield. Fortunately, the incorporation of MoO_4_‐CoNi LDH onto CuO NW/CF effectively addresses this limitation (Figure 2b,c; Figure S18, Supporting Information). The selectivity results for NO_2_^−^ and NH_3_ at different potentials are depicted in Figure S19 (Supporting Information), exhibiting a remarkable selectivity of up to 96.39% for NH_3_ at −0.2 V vs. RHE.

To further validate the origin of synthesized NH_3_ from injected NO_3_^−^, a ^15^N isotope labeling experiment was conducted. Following electrolysis at −0.2 V vs. RHE using Na^15^NO_3_ and Na^14^NO_3_ as reactants, the ^1^H nuclear magnetic resonance (^1^H NMR, 400 MHz) spectra of the electrolyte exhibited characteristic bimodal peaks for ^15^NH_4_^+^ and trimodal peaks for ^14^NH_4_^+^, respectively.^[^ ^12^ ^]^ This isotope labeling experiment conclusively demonstrates that the generated NH_3_ originates solely from NitRR, effectively excluding any potential influence from external environmental pollutants or electrocatalyst interference (Figure 2d).

To evaluate MoO_4_‐CoNi LDH/CuO NW/CF's capability in nitrate removal, a batch conversion test was conducted using an initial concentration of ≈0.05 mol L^−1^ NO_3_^−^ solution, followed by measurement of the residual product. After 2 h of electrolysis, nearly all of the nitrogen sources were efficiently converted into NH_3_ within the same time frame, and both NO_3_^−^ and NO_2_^−^ concentrations remained well below the drinking water regulations set by the World Health Organization (WHO) (Figure 2e). To further highlight their industrial production potential, a series of experiments were performed. First, electrolytes with different NO_3_^−^ concentrations (0.005‐0.2 mol L^−1^) were studied, and the high performance was found to be maintained well over a wide range (Figure 2f).^[^ ^28^ ^]^ Across seven consecutive cycles, the variations in NH_3_ FE and current density of MoO_4_‐CoNi LDH/CuO NW/CF remain consistent, indicating the excellent stability of MoO_4_‐CoNi LDH/CuO NW/CF during the electrocatalytic nitrate‐to‐ammonia conversion process (Figure 2g). In fact, after the electrochemical tests, the XPS spectra of copper, nickel, cobalt and molybdenum showed almost no change. Meanwhile, the metal ions in the solution only had a small amount of residual after the first cycle, indicating that it has extremely high durability (Figures S20 and S21, Supporting Information). Figure 2h compares the nitrate reduction performance of MoO_4_‐CoNi LDH/CuO NW/CF with that of other electrocatalysts, and the detailed comparison of nitrate reduction performance is summarized in Table S3 (Supporting Information). MoO_4_‐CoNi LDH/CuO NW/CF exhibits a high FE for NH_3_ production at high partial current densities, which is superior or comparable to the performance of non‐noble metal or alloy catalysts.^[^ ^12^, ^16^, ^25^, ^29^, ^30^, ^31^, ^32^, ^33^, ^34^ ^]^

### 2.3 Kinetics Evaluation of Catalytic Tandem Reactions

The above results depict a two‐step tandem reaction, wherein the reactant concentration (NO_3_^−^) gradually diminishes over the course of the reaction, while the product concentration (NH_3_) steadily increases. Additionally, the concentration of intermediates (NO_2_^−^) initially rises and then declined, reaching its maximum value in the middle. The kinetic characteristics of this reaction indicate that both elementary reactions occur simultaneously; once an intermediate (NO_2_^−^) is formed, its transformation into a product (NH_3_) promptly commences. The relative concentrations of reactants (NO_3_^−^), intermediates (NO_2_^−^), and products (NH_3_) depend on the relative rates of these two‐step tandem reactions. Catalytic tandem reactions pose challenges for catalyst optimization and reaction condition design due to their interdependence on two distinct reactions occurring concurrently. Consequently, it becomes difficult to independently tailor each reaction's characteristics and properties or achieve precise control over them. Therefore, we analyze reactant reduction rate in solution and product formation rate by examining reactants, intermediates, and products to accomplish meticulous regulation of the reaction kinetics. From the trend of nitrogen concentration changes during the high‐concentration (100 mm) nitrate reaction process, it can be seen that our catalyst system is a typical two‐step tandem reactions (Figure S22, Supporting Information).

Nitrate reduction is visualized as a nitrogen species reduction and hydrogenation process, resulting from the chemical reactions of direct nitrogen reduction and hydrogenation of reactive hydrogen on the catalyst surface, occurring at two distinct sites. The process of nitrate reduction to ammonia can be divided into two main steps: nitrate reduction to nitrite and nitrite reduction to ammonia. The kinetic rate constants are denoted as *k*_1_ and *k*_2_, respectively, and the corresponding formulas is presented below (Equations 2 and 3):

(2) $${\mathrm{NO}}_{3}^{-}+H_{2}O+2e^{-}\overset{k_{1}}{\to }{\mathrm{NO}}_{2}^{-}+2OH^{-}$$

(3) $${\mathrm{NO}}_{2}^{-}+5*H+6e^{-}\overset{k_{2}}{\to }NH_{3}+2OH^{-}$$

**Figure 3:**
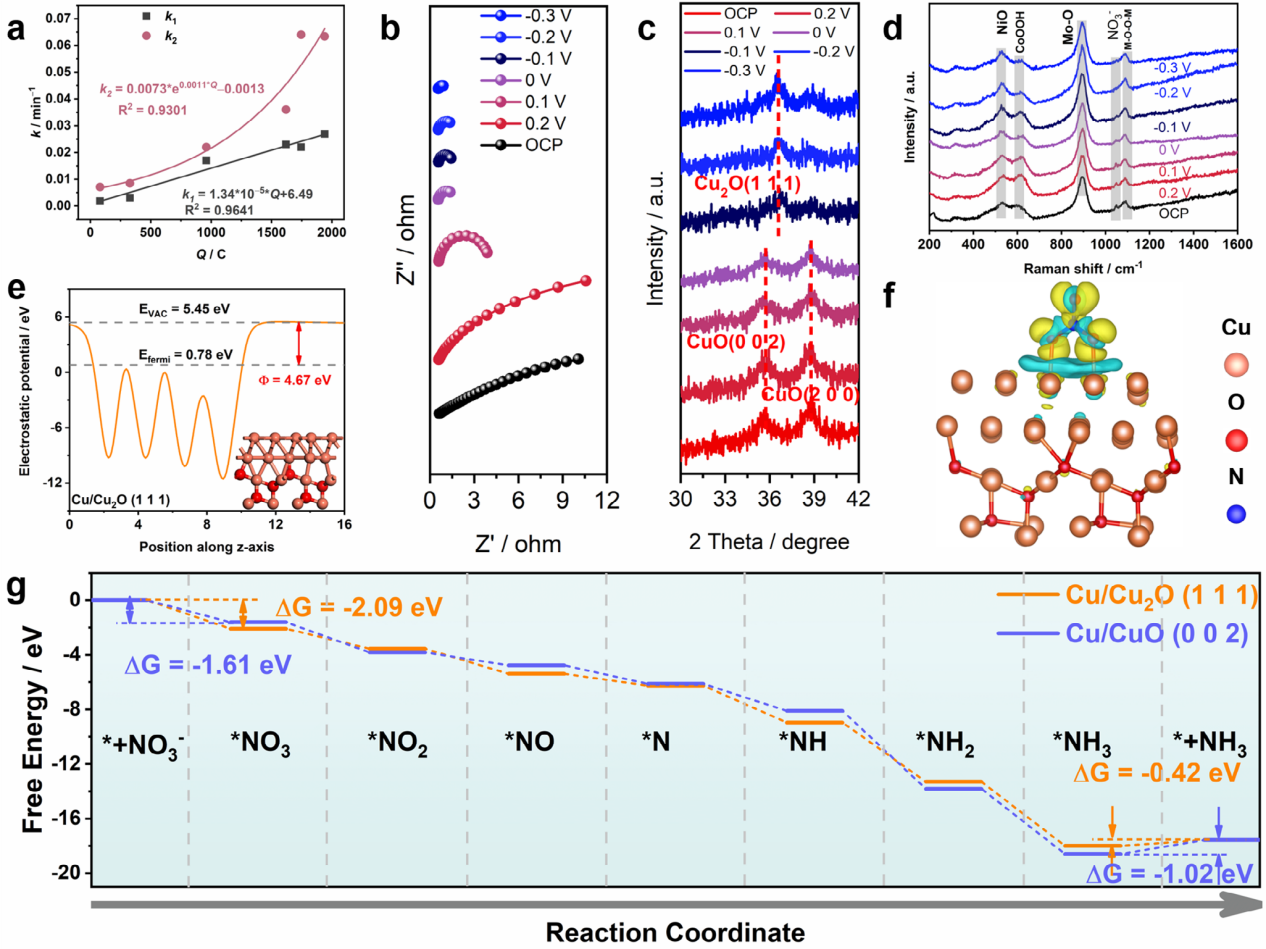
Mechanism of nitrate catalysis by copper‐based catalysts. a) The relationship between the kinetic constant (*k*_1_ and *k*_2_) of the reaction and *Q* of the reaction. b) Nyquist plot of CuO NW/CF at various potentials of 1 mol L^−1^ KOH (0.05 mol·L^−1^ NO_3_^−^). c) Ex situ XRD spectra of the CuO NW/CF catalyst at different applied potentials in 1 mol L^−1^ KOH electrolyte with 0.05 mol L^−1^ NaNO_3_. d) In situ Raman spectra of the MoO_4_‐CoNi LDH/CuO NW/CF at different applied potentials in 1 mol L^−1^ KOH electrolyte with 0.05 mol L^−1^ NaNO_3_. e) Electrostatic potentials of Cu/Cu_2_O (1 1 1) surface. f) Charge density difference illustrating *NO_3_ adsorption on Cu/Cu_2_O (1 1 1) surface. g) Calculated Gibbs free energy changes for NitRR leading to NH_3_ production at 0 V vs. RHE.

Previous findings have demonstrated the remarkable catalytic activity of CuO in facilitating the conversion of nitrate to nitrite.^[^ ^29^ ^]^ In order to investigate this further, we conducted a series of nitrate conversion experiments at varying voltages and subsequently determined the corresponding rate constants. The results indicate that while there was no significant alteration observed in *k*_1_, a decrease in voltage led to an increase in *k*_2_. The primary reason for this phenomenon is that the electrode potential for nitrate reduction to nitrite is 0.836 V vs. RHE (Equation 4), whereas the electrode potential for nitrite reduction to ammonia is 0.716 V vs. RHE (Equation 5), resulting in a preference for nitrate reduction to nitrite (*k*_1_) at high potentials.^[^ ^13^ ^]^ While *k*_1_ remains relatively constant as the potential decreases, *k*_2_ continues to increase until reaching rate matching at maximum Faraday efficiency (Figure S23, Supporting Information). The decrease in the potential results in an increase in the reaction charge, thereby inducing an upward trend in both *k*_1_ and *k*_2_. By fitting the relationship between *k*_1_ and *k*_2_ against the amount of charge, we observed a linear correlation between *k*_1_ and quantity of electric charge (*Q*), while an exponential correlation was found between *k*_2_ and *Q*, indicating that changes in *Q* had a greater impact on *k*_2_ than on *k*_1_. Upon conversion, it was evident that there existed an exponential correlation between *k*_2_ and *k*_1_ (**Figure** **3**a).

(4) $$N{O_{3}}^{-}+H_{2}O+2e^{-}\to \;N{O_{2}}^{-}+2OH^{-}\;\;E^{0}=0.836\;V\;\mathrm{vs}.\;\mathrm{RHE}$$

(5) $$N{O_{2}}^{-}+5H_{2}O+6e^{-}\to \;NH_{3}+7OH^{-}\;\;E^{0}=0.716\;V\;\mathrm{vs}.\;\mathrm{RHE}$$

To investigate the exceptional catalytic activity of CuO NW/CF on NO_3_^−^, CuO NW/CF was pre‐reduced at −0.2 V vs. RHE for 30 min and the pre‐reducing electrode was immersed in an electrolyte solution (including 1 mol L^−1^ KOH and 0.05 mol L^−1^ NaNO_3_), where nitrate was converted to nitrite by CuO NW/CF without voltage applied. This observation suggests a spontaneous redox reaction between CuO or its reduction products and NO_3_^−^, resulting in the generation of NO_2_^−^. Simultaneously, this polarization phenomenon leads to a decrease in the open circuit voltage (Figure S24, Supporting Information).

Electrochemical impedance spectroscopy (EIS) is a potentially valuable experimental technique for investigating the kinetics of electrocatalytic reactions and the properties of the electrode/electrolyte interface. In order to gain a deeper understanding of the reaction kinetics in 1 mol L^−1^ KOH and 0.05 mol L^−1^ NaNO_3_ solutions, in situ EIS measurements were conducted. Figure 3b depicts the measured impedance spectra of the NitRR process on CuO NW/CF within an electrode potential range of 0.2 to −0.3 V vs. RHE, while equivalent circuits are used to fit Nyquist plots of these impedance spectra (Figure S25, Supporting Information). The equivalent circuit comprises a solution resistance (*R*_s_), a constant phase element (CPE), and a charge transfer resistance (*R*_ct_). The results obtained from fitting EIS data are presented in Table S4 (Supporting Information). *R*_ct_ and CPE can reflect the adsorption behavior of reactants or intermediates on the catalyst surface. Since CuO NW/CF exhibits similar *R*_s_ values at several voltages, *R*_ct_ can serve as an indicator for electrode resistance. As potential decreases, *R*_ct_ gradually diminishes for CuO NW/CF, while Cu_2_O begins to form progressively faster. These findings indicate that Cu_2_O formation involves more rapid electron transfer and accelerated adsorption kinetics for reactants or intermediates during NitRR process.

In order to elucidate the mechanism underlying the reduction of NO_3_^−^ to NO_2_^−^, ex situ XRD tests were performed on CuO NW/CF following pre‐reduction. The experimental findings demonstrated that during open circuit potential (OCP) conditions, alongside the presence of a Cu peak, an additional signal indicative of CuO was observed. Upon gradual reduction in potential from 0.2 down to 0 V vs. RHE, there was a decline in intensity for the CuO signal until its near disappearance; concurrently at the potential (0 V vs. RHE), the distinct feature associated with Cu_2_O became evident (Figure 3c). Moreover, as further decrease in potential occurred from 0 V toward −0.3 V vs. RHE, there was a progressive enhancement observed for the intensity of this newly formed Cu_2_O signal (Equations (6), (7), (8)).

For the study on phase transition of LDH, Figure 3d demonstrates the variation of several peaks in Raman spectra with the decrease of potential. The peaks at 530 cm^−1^ and 600 cm^−1^ primarily arise from the A_1__g_ tensile vibration mode of Ni‐O and CoOOH, with the region between the Ni‐O and CoOOH peaks gradually expanding as voltage decreases, attributed to enhanced availability of hydrogen radicals at lower voltages.^[^ ^35^, ^36^ ^]^ Furthermore, a decrease in nitrate peak (1047 cm^−1^) is observed as the voltage decreases, mainly due to nitrate degradation under low voltage conditions.

(6) $$\mathrm{CuO}+2e^{-}+H_{2}O\;\to \;\mathrm{Cu}+2OH^{-}$$

(7) $$2\mathrm{CuO}+2e^{-}+H_{2}O\;\to \;Cu_{2}O+2OH^{-}$$

(8) $$2\mathrm{Cu}+N{O_{3}}^{-}\;\to \;Cu_{2}O+N{O_{2}}^{-}$$

Density functional theory (DFT) calculations have been widely used to study the reaction paths and related mechanisms of the NitRR. Therefore, DFT calculations were employed here to gain a comprehensive understanding of the underlying mechanisms responsible for the notable variations in electrocatalytic activity resulting from the incorporation of diverse heteroatoms in Cu/Cu_2_O and acid group anion‐CoNi LDH. The work function of a surface is a significant criterion for investigating the surface activity. The work functions of the Cu/CuO (0 0 2) and Cu/Cu_2_O (1 1 1) surfaces were calculated based on the following (Equation 9):

(9) $$\Phi =E_{\mathrm{VAC}}\;-\;E_{\mathrm{fermi}}$$

where *E*_fermi_ is the Fermi energy, and *E*_VAC_ is the electrostatic potential of the vacuum level. The work functions of the Cu/CuO (0 0 2) and Cu/Cu_2_O (1 1 1) surfaces were 7.43 eV (Figure S26, Supporting Information) and 4.67 eV (Figure 3e), respectively. Comparing the work functions of the Cu/CuO (0 0 2) and Cu/Cu_2_O (1 1 1) surface, electrons are more likely to overflow from the Cu/Cu_2_O (1 1 1) surface, which is more active than the Cu/CuO (0 0 2) surface. Thus, Cu/Cu_2_O (1 1 1) is more conducive to the initial *NO_3_ adsorption. Moreover, the charge density difference calculation results for NO_3_^−^ before and after adsorption at the Cu/Cu_2_O (1 1 1) (*Cu/Cu_2_O (1 1 1)) (Figure 3f) reveal a transfer of oxygen atoms from NO_3_^−^ to the Cu position at the Cu/Cu_2_O (1 1 1), providing strong evidence for enhanced interaction between *NO_3_ and regions of the Cu/Cu_2_O (1 1 1).

The overall NitRR pathway on the catalyst surface is shown in Figure 3g and the related structures of intermediates are shown in Figure S27 (Supporting Information). The pathway includes the adsorption of NO_3_^−^ to form *NO_3_, deoxygenation of the N species, hydrogenation of the N species, and desorption of the reduced species. It is clear that the Gibbs adsorption energy (*G*_ads_) of *NO_3_ on Cu/Cu_2_O (1 1 1) is −2.09 eV, lower than that of Cu/CuO (0 0 2) (−1.61 eV), suggesting that the Cu/Cu_2_O (1 1 1) facet triggers the reaction. The adsorption energy of other intermediates (*NO_2_, *NO, *N, *NH, *NH_2_, *NH_3_) on the Cu/CuO (0 0 2) surface is higher than that of Cu/Cu_2_O (1 1 1). However, previous studies have indicated that the catalyst's robust adsorption of NitRR intermediates (*NO_2_, *NO, *N, *NH, *NH_2_, *NH_3_) often leads to rapid deactivation, thereby impeding subsequent electrochemical reduction into NH_3_.^[^ ^9^ ^]^ In addition, the NH_3_ desorption step (*NH_3_+e^−^→NH_3_) from Cu/CuO (0 0 2) exhibits a higher uphill Δ*G* of 1.02 eV compared to the same reaction step of NH_3_ desorption from Cu/Cu_2_O (1 1 1) which only requires 0.42 eV. The aforementioned analysis results indicate that NO_3_^−^ adsorption, intermediate adsorption, and NH_3_ desorption processes on Cu/Cu_2_O (1 1 1) outperform that of Cu/CuO (0 0 2).

**Figure 4:**
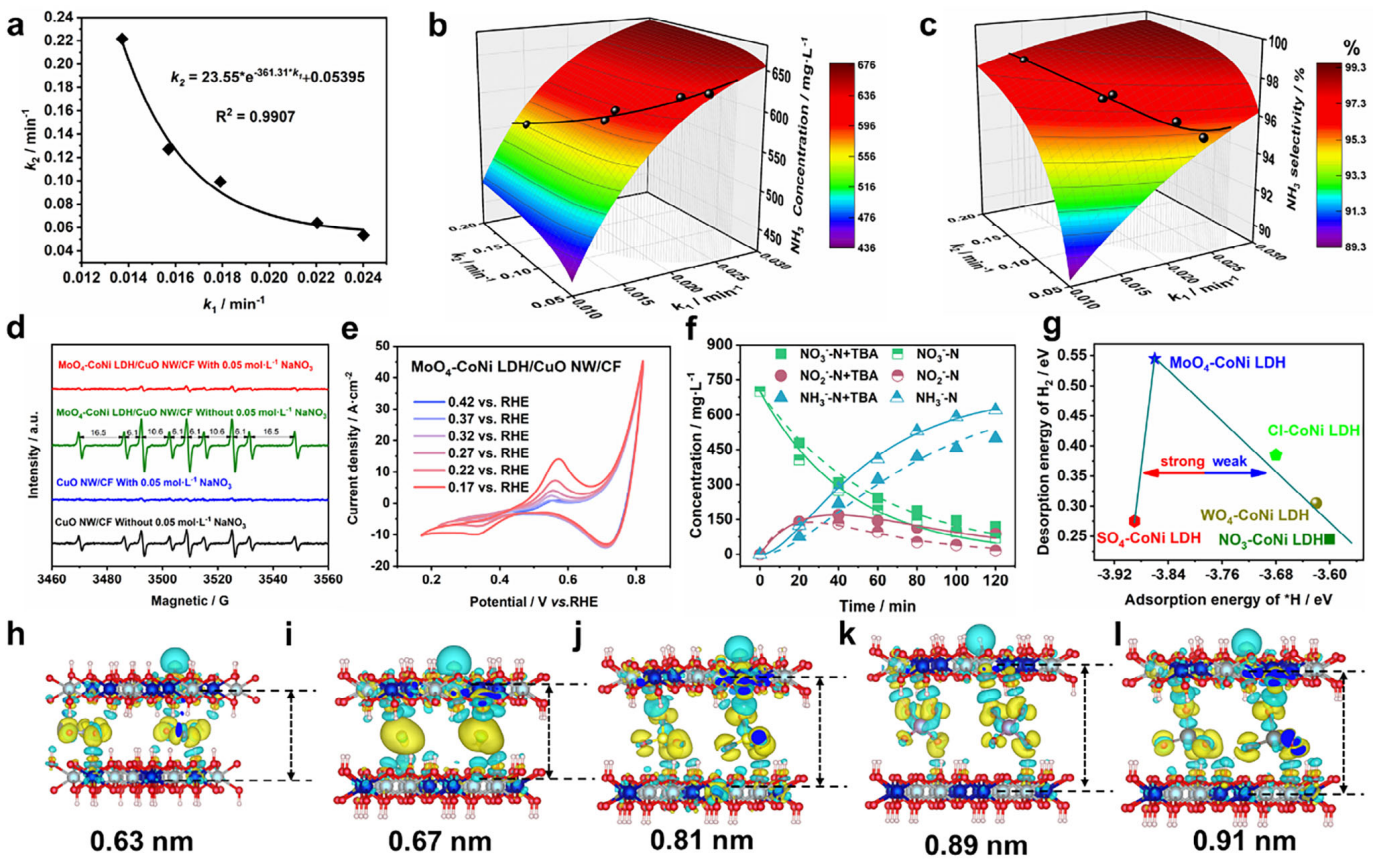
Mechanism of hydrogen radicals supply catalyzed by CoNi LDH. a) Correlation between reaction rate constants *k*_2_ and *k*_1_ for MoO_4_‐CoNi LDH/CuO NW/CF with varying anion intercalations under identical voltage conditions. 3D colormap surface plot and corresponding colortour maps for of NH_3_ concentration b) and NH_3_ selectivity c) on the rate constant of nitrite reduction by different nitrates (*k*_1_) and the rate constant of nitrite reduction to ammonia (*k*_2_). d) Electrochemical quasi in situ EPR tests at −0.2 V versus RHE. e) Effects of starting potential on CV curves of MoO_4_‐CoNi LDH/CuO NW/CF in Ar‐saturated 1 mol L^−1^ KOH solution. f) Under TBA masking conditions, the MoO_4_‐CoNi LDH/CuO NW/CF electrode exhibited a NO_3_^−^ change over time in a −0.2 V versus RHE, 1 mol L^−1^ KOH (0.05 mol L^−1^ NaNO_3_) solution. g) The correlation between the rate constant of nitrite reduction to ammonia (*k*_2_) and the activation energy for *H was investigated. Differences in charge density adsorbed on the surface of CoNi LDH with varying anion intercalations. h–l) Variations in charge density of hydrogen radicals adsorbed on the surface of CoNi LDH with different anion intercalations.

To gain a more comprehensive understanding of the impact of CoNi LDH loading on the hydrogenation process, electron paramagnetic resonance (EPR), radical masking, cyclic voltammetry (CV) tests, and DFT calculations were employed to investigate the generation and transfer of hydrogen radicals (*H). To investigate the impact of *H on reaction kinetics, we conducted kinetic analysis of CoNi LDH/CuO NW/CF intercalated with various anions (Cl^−^, NO_3_^−^, SO_4_^2−^, MoO_4_^2−^, WO_4_^2−^). The results revealed an increasing trend in *k*_1_ while a decreasing trend in *k*_2_ as the layer spacing increased. The findings from the aforementioned studies demonstrate an exponential correlation between variations in *k*_2_ and *k*_1_. By studying the kinetics of CoNi LDH/CuO NW/CF tandem reactions with different anion intercalations, it was observed that *k*_2_ gradually decreased with increasing CoNi LDH layer spacing. The fitting results presented above demonstrate an exponential correlation between *k*_1_ and *k*_2_ when the charge input is in close proximity (**Figure** **4**a; Figure S28, Supporting Information).

To investigate the impact of changes in *k*_1_ and *k*_2_ on NO_2_^−^ concentration, NH_3_ concentration, and NH_3_ selectivity, we initially set the NO_3_^−^ concentration at 700 mg L^−1^ and the reaction time at 120 min as baseline values. In order to effectively separate and enhance the tandem catalytic process, we developed a kinetic‐descriptor tool based on the widely accepted mechanism of electrocatalytic ammonia nitrate synthesis for studying electrocatalytic nitrate synthesis of ammonia. Subsequently, we derived the following equations to determine three kinetic descriptors and visually depicted the relationship between *k*_1_, *k*_2_, NO_2_^−^ concentration, NH_3_ concentration, and NH_3_ selectivity using 3D color plots. The equation is represented as follows (Equations (10), (11), (12)):

(10) $$C\left({\mathrm{NO}}_{2}^{-}\right)=\frac{700k_{1}}{k_{1}-k_{2}}\left(e^{-120k_{2}}-e^{-120k_{1}}\right)$$

(11) $$C\left({\mathrm{NH}}_{3}\right)=700\left(1+\frac{k_{1}e^{-120k_{2}}-k_{2}e^{-120k_{1}}}{k_{2}-k_{1}}\right)\;$$

(12) $$S\left(NH_{3}\right)/\%=\frac{C\left(NH_{3}\right)}{C\left({\mathrm{NO}}_{2}^{-}\right)+C\left(NH_{3}\right)}$$

The accumulation of NO_2_^−^ increased gradually as *k*_2_ decreased, primarily due to the decrease in *k*_2_ resulting in reduced NO_2_^−^ consumption and ultimately leading to the buildup of NO_2_^−^ (Figure S29, Supporting Information). However, as illustrated in Figure 4b, the increase in *k*_1_ and *k*_2_ also leads to a higher NH_3_ yield. Nevertheless, compared to the impact of *k*_2_ on NH_3_ yield, changes in *k*_1_ have a more pronounced effect. This observation further supports the idea that nitrate reduction to nitrite acts as the rate‐determining step during the process of nitrate reduction to ammonia. Moreover, both *k*_1_ and *k*_2_ exhibit a positive correlation with NH_3_ selectivity and yield. Although increasing these rate constants enhances NH_3_ production and selectivity, our findings indicate that they are influenced by factors such as input charge amount and electrode material. By incorporating the relationship between *k*_1_ and *k*_2_ for different layer spacings mentioned above into Figures S22 (Supporting Information), it can be observed that NO_2_^−^ and NH_3_ yields gradually increase with increasing layer spacing; however, this results in a decrease in NH_3_ selectivity. Therefore, theoretically speaking, MoO_4_‐CoNi LDH represents an optimal choice for achieving maximum NH_3_ yield while maintaining high selectivity (Figure 4b,c).

Notably, EPR results (Figure 4d) revealed that only a small amount of *H was generated in solution during the electrocatalysis of nitrate by CuO NW/CF. In contrast, when MoO_4_‐CoNi LDH was loaded onto CuO NW/CF, the peak intensity surpassed that of CuO NW/CF, indicating an enhanced *H supply capacity due to MoO_4_‐CoNi LDH loading. The CV curve shown in Figure 4e and Figure S30 (Supporting Information) also exhibited an oxidation peak corresponding to *H; however, this oxidation peak for MoO_4_‐CoNi/CuO NW/CF was significantly higher than that observed for CuO NW/CF. To confirm the crucial role of *H in the electrochemical system, *tert*‐butyl alcohol (TBA), a specific quenching agent for *H, was added prior to electrolysis. There was reduced removal efficiency for NO_3_^−^ and NO_2_^−^ as it hindered their reduction by *H with TBA. The MoO_4_‐CoNi LDH/CuO NW/CF catalyst demonstrated a reduction effect on 1 mol L^−1^ KOH (with NO_3_^−^) solution; consequently, *k*_1_ and *k*_2_ rate constants decreased from 0.022 to 0.017 h^−1^ and from 0.064 to 0.036 h^−1^ respectively when TBA was added to the solution. The rate constant for reduction of NO_2_^−^ to NH_3_ decreased approximately by a factor of one providing evidence supporting the critical role played by *H as an intermediate reaction species in reducing NO_2_^−^ into NH_3_ (Figure 4f). Without TBA, when the potential is lower than −0.3 V vs. RHE, the cathode current rises sharply, mainly driven by the nitrate reduction reaction (NitRR). After adding TBA, the total current density shows a trend of first decreasing and then increasing. The first decrease is due to the adsorption of TBA at the *H generation site, which partially inhibits the generation of *H. As the voltage further decreases, the voltage can drive the breakthrough of TBA's limitation, and the TBA in the solution will consume *H, resulting in an increase in current density (Figure S31, Supporting Information).

The primary role of CoNi LDH is to serve as a hydrogen radical (*H) provider (Figure S32, Supporting Information). Initially, water molecules are adsorbed onto the surface of CoNi LDH, followed by detachment of OH^−^ from CoNi LDH to form *H. *H absorbed on the surface of CoNi LDH can either combine with other *H to form H_2_ or directly for NitRR. The release barriers of *H and H_2_ serve as reasonable descriptors of the competitive relationship between NitRR and HER. A higher Δ*G*_H2_ value suggests weak HER activity in the catalyst, which favors the NitRR reaction. We systematically screened CoNi LDH catalysts with various anionic intercalations and calculated the energy barriers for *H and H_2_ release during the HER process. For efficient nitrite reduction to ammonia in the NitRR process, a moderate *H release barrier is crucial. If it is too strong, it may poison the CoNi LDH catalyst, while if it is too weak, undesired desorption of *H leading to by‐products like H_2_ can occur. Notably, we observed a volcano‐like correlation between the binding energy of *H and that released by H_2_. The MoO_4_‐CoNi LDH exhibits a moderate binding energy toward *H along with a high energy barrier for H_2_ release, making it a promising candidate as a hydrogen radical donor (Figure 4g; Figure S33, Supporting Information).

The charge density difference of H before and after adsorption at the CoNi LDH interface is illustrated in Figure 4h–l, demonstrating the occurrence of charge transfer between oxygen and hydrogen atoms. Variations in anion intercalations induce changes in the layer spacing of CoNi LDH, which consequently affects its surface charge. Consequently, alterations in surface charge can impact the bond lengths of adatoms as well. Notably, an increase in layer spacing exhibits a trend where the surface H‐O bond length initially increases and then decreases within CoNi LDH due to distinct anions' charge contributions to this material. Accumulation of charges on the CoNi LDH layer leads to shorter hydrogen/oxygen bond lengths, thereby promoting hydrogen radical production and highlighting MoO_4_‐CoNi LDH's advantage for this purpose.

### 2.4 Rechargeable Zn‐Nitrate Battery and Ammonia Product Collection

**Figure 5:**
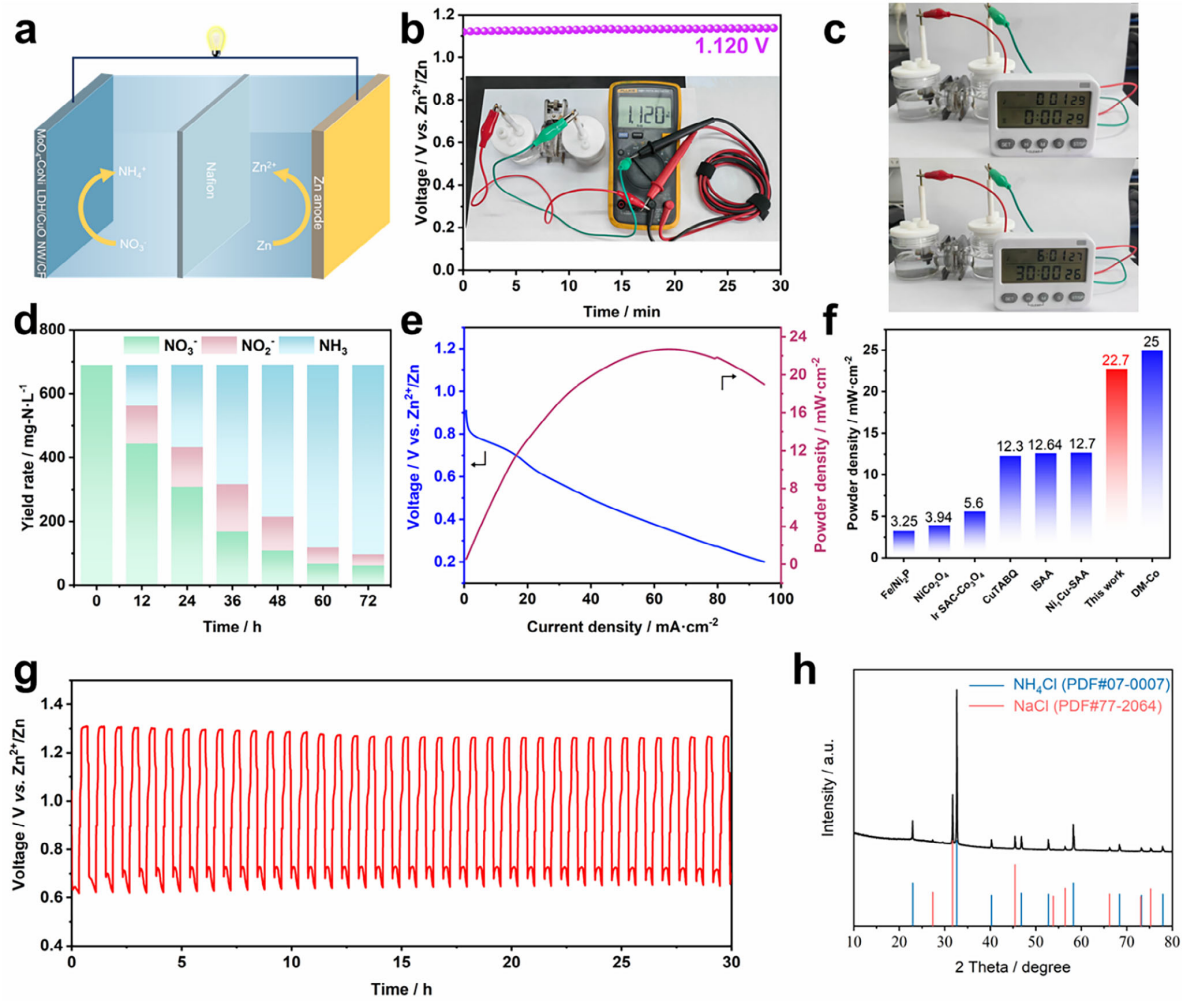
The electrochemical performance of hybrid aqueous Zn‐NO_3_ battery. a) Schematic representation of the assembled Zn‐NO_3_ battery featuring a Zn anode and MoO_4_‐CoNi LDH/CuO NW/CF cathode. b) Open circuit voltage of Zn‐NO_3_ battery. c) Visual representation with photographs showcasing a timepiece powered by the Zn‐NO_3_ battery. d) Long‐term discharge stability test results for the Zn‐NO_3_ battery at 20 mA cm^−2^, including the corresponding yield. e) Discharging polarization curves and corresponding power density curves of the Zn‐NO_3_ battery. f) Comparative analysis of electrochemical performance with benchmark reported catalysts (See detailed information in Table S5, Supporting Information). g) Galvanostatic discharge–charge cycling curves over 40 cycles at 12.5 mA cm^−2^. h) Synthesized NH_4_Cl products and corresponding XRD patterns.

The schematic diagram of the aqueous rechargeable zinc‐nitrate (Zn‐NO_3_) battery is illustrated in **Figure** **5**a. During discharge, Zn metal on the anode dissolves and releases electrons, facilitating NitRR at the cathode through electron transfer. On charging, water undergoes oxidation to produce O_2_, while Zn (OH)_4_^2−^ is formed at the anode leading to the generation of Zn, thereby resulting in the subsequent battery reaction.

Discharge reaction:

(13) $$\begin{array}{ccc} & & 4\mathrm{Zn}+N{O_{3}}^{-}+7H_{2}O+6OH^{-}\to 4\mathrm{Zn}{{\left(\mathrm{OH}\right)}_{4}}^{2-}\\ & & +\mathrm{NH}4^{+},\;E_{\mathrm{discharge}}=1.152\;V \end{array}$$

Charge reaction:

(14) $$2\mathrm{Zn}{{\left(\mathrm{OH}\right)}_{4}}^{2-}\to 2\mathrm{Zn}+2H_{2}O+4OH^{-}+O_{2},\;E_{\mathrm{charge}}=1.650\;V$$

Overall reaction:

(15) $$N{O_{3}}^{-}+3H_{2}O\to N{H_{4}}^{+}+2OH^{-}+2O_{2}$$

The constant open circuit potential of MoO_4_‐CoNi LDH/CuO NW/CF‐based Zn‐NO_3_ battery is 1.12 V vs. Zn/Zn^2+^, as depicted in Figure 5b. This value closely aligns with the theoretical voltage of 1.15 V vs. Zn/Zn^2+^ for the Zn‐NO_3_ battery. The battery was utilized to power an electronic timer, and the device operated continuously for 24 h without any anomaly (Figure 5c).

Typically, the voltage of Zn‐NO_3_ battery stays ≈0.83 V at 10 mA cm^−2^, which lowers the calculated energy density to 634.88 Wh L^−1^ with 3.76 mol L^−1^ of NO_3_^−^ in the electrolyte (Figure S34, Supporting Information). The conversion of nitrate to ammonia exceeds 90% within a duration of 72 h, as depicted in Figure 5d, under a current density of 20 mA cm^−2^. As a result, the Zn‐NO_3_ battery reaches a maximal power density of 22.7 mW cm^−2^, which is superior to the reported catalysts (Figure 5e,f; Table S5, Supporting Information).^[^ ^37^, ^38^, ^39^, ^40^, ^41^, ^42^, ^43^ ^]^ Furthermore, continuous discharge–charge cycling curves at 12.5 mA cm^−2^ show good stability and a low charge potential of 1.30 V during 40 cycles (Figure 5g), which suggests excellent stability of the assembled Zn‐NO_3_ battery.

We further demonstrate the practical application of our approach by integrating electrocatalysis with gas extraction to achieve continuous collection of high‐purity ammonia products (Figure S35, Supporting Information). More than 95% of ammonia can be effectively removed by the air stripping method, followed by neutralization of the resulting HCl solution (containing NH_3_) using NaOH and subsequent rotary evaporation. Approximately 80.1% of NH_3_ is converted into NH_4_Cl powder. XRD analysis confirmed the formation of NH_4_Cl and NaCl (from NaOH neutralization) compounds (Figure 5h). In summary, we have presented an integrated process for directly converting nitrate‐containing feedstock water into high‐quality ammonia products using a MoO_4_‐CoNi LDH/CuO NW/CF catalyst.

## 3 Conclusion

In summary, we report a highly efficient strategy for nitrate reduction to ammonia using anion‐intercalated CoNi LDH/CuO NW/CF catalysts. By tuning the anion intercalation (Cl^−^, NO_3_^−^, SO_4_^2−^, MoO_4_^2−^, WO_4_^2−^) and modulating the layer spacing in CoNi LDH, we enhanced *H generation and facilitated the hydrogenation of nitrogenous intermediates, significantly improving catalytic performance. The MoO_4_‐CoNi LDH/CuO NW/CF catalyst achieved an ammonia yield of 1.12 mmol cm^−2^ h^−1^ and a Faraday efficiency of 99.78% at an ultra‐low potential of −0.2 V vs. RHE, outperforming conventional copper‐based catalysts. In situ spectroscopic and electrochemical studies revealed that Cu/Cu_2_O redox processes drive the reduction of nitrate to nitrite, while CoNi LDH acts as a key *H donor, accelerating nitrite hydrogenation to ammonia. Tandem reaction kinetics, described by rate constants (*k_1_* for nitrate‐to‐nitrite and *k_2_* for nitrite‐to‐ammonia) and a volcano curve model, provide a comprehensive understanding of the reaction pathway and enable the rational selection of optimal catalysts. These insights pave the way for the design of more efficient catalysts for sustainable ammonia production and nitrate removal.

## Conflict of Interest

The authors declare no conflict of interest.

## Supporting information



Supporting Information

## Data Availability

Research data are not shared.
