# Supplementary material for: Two‐Step Tandem Catalysis for High‐Efficiency Ammonia Synthesis Via Nitrate Reduction on Anion‐Intercalated CoNi LDH and Cu/Cu2O
Source: Adv Sci (Weinh). 2025 Apr 15;12(26):2502262. doi: 10.1002/advs.202502262 (PMC12245002; doi:10.1002/advs.202502262)
Supplement: Supplementary file 1 — Supporting Information [file ADVS-12-2502262-s001.docx]

Supporting Information

Two-Step Tandem Catalysis for High-Efficiency Ammonia Synthesis via Nitrate Reduction on Anion-intercalated CoNi LDH and Cu/Cu_2_O

*Changzheng Lin^[^**^a]^, Weijia Li^[a]^, Hao Chen^[a]^, Jiangtao Feng*^[a]^, Mengyuan Zhu^[c]^, Jinwen Shi^[c]^, Mingtao Li^[c]^, Bo Hou*^[b]^, Zhenyu Wang^[a]^, Xin Chen^[a]^, Jia Liu^[d]^, Wei Yan*^[a], [c]^*

[a] CZ Lin, WJ Li, H Chen, JT Feng, ZY Wang, X Chen, W Yan
Department of Environmental Science & Engineering, School of Energy and Power Engineering, Xi’an Jiaotong University, 28 Xianning West Road, Xi’an, 710049, China.
Email: fjtes@xjtu.edu.cn (Jiangtao Feng)

[b] B Hou
School of Physics and Astronomy, Cardiff University, The Parade, Cardiff, CF24 3AA, Wales, UK.Department,
HouB6@cardiff.ac.uk (Bo Hou)

[c] MY Zhu, JW Shi, MT Li, W Yan
International Research Center for Renewable Energy (IRCRE), State Key Laboratory of Multiphase Flow in Power Engineering, School of Energy and Power Engineering, Xi’an Jiaotong University, 28 Xianning West Road, Xi’an 710049, China.
yanwei@xjtu.edu.cn (Wei Yan)

[d] J Liu
Instrument Analysis Center of Xi'an Jiaotong University, Xi’an Jiaotong University, Xi’an 710049, China

Corresponding authors:

Jiangtao Feng: fjtes@xjtu.edu.cn

Bo Hou: HouB6@cardiff.ac.uk

Wei Yan: yanwei@xjtu.edu.cn

Table of Contents

[**1** **Experimental Procedures** 3](#_Toc195024655)

[**2 Supporting Figures** 11](#_Toc195024656)

[**3 Supporting Tables** 25](#_Toc195024657)

[**4 Supporting References** 27](#_Toc195024658)

1. **Experimental Procedures**
   1. **Materials**

*Chemicals:* Sodium hydroxide (NaOH, AR, ≥96%), ammonium persulfate ((NH_4_)_2_S_2_O_8_, AR, ≥98.0%), nickel(II) nitrate (Ni(NO_3_)_2_, AR, ≥98%), hydrochloric acid (HCl, AR, 36%~38%), sulfuric acid (H_2_SO_4_, AR, 95%~98%), sodium nitrate (NaNO_3_, AR, ≥99.0%), cobalt(II) sulfate (CoSO_4_, AR, ≥99.5%), nickel(II) sulfate (NiSO_4_, AR, ≥98.5%), cobalt(II) chloride (CoCl_2_, AR, ≥99.0%), nickel(II) chloride (NiCl_2_, AR, ≥98.0%), sodium nitrite (NaNO_2_, AR, ≥99.0%), tert-butanol (C_4_H_10_O, AR, ≥99.5%), and zinc acetate (Zn(CH_3_COO)_2_, AR, ≥99.0%) were purchased from Sinopharm Chemical Reagent Co., Ltd. (Beijing, China). Cobalt(II) nitrate (Co(NO_3_)_2_, AR, ≥99%), sodium molybdate (Na_2_MoO_4_, AR, ≥99%), and Sodium tungstate dihydrate (Na_2_WO_4_, AR, ≥99.5%) were purchased from Shanghai Aladdin Biochemical Technology Co., Ltd. Except noted, all chemicals were purchased and used without further purification. All solutions were prepared using distilled water (DIW) with an electrical conductivity of 18.25 MΩ.

Copper foam (CF) was purchased from Xi'an Modeke Trading Co. Ltd, (Xi’an, China). Product parameters of CF: pore size (0.1 mm-10 mm), porosity (50%-98%), through porosity (≥98%), volume density (0.1-0.8 g·cm^−3^) and thickness (1 mm). The CF were sequentially cleaned by DIW, ethanol and 1 M HCl for 15 min in an ultrasonic bath. Finally, these substrates were dried in ambient air.

- 1. **Synthesis method**

Preparation of Cu(OH)_2_ NW/CF: In a typical procedure, 1 × 2 cm^2^ Cu foam (CF) was first washed with isopropyl alcohol and then followed sonication in 0.1 M HCl aqueous solution for 15 min to clean the surface. Next, the washed CF was soaked in a mixture solution with 0.125 M (NH_4_)_2_S_2_O_8_ and 2.5 M NaOH at 25^o^C for 15 min to oxidize Cu into Cu(OH)_2_ nanowire (NW)/CF. After the chemical oxidation reaction, the Cu(OH)_2_ NW/CF was rinsed with deionized water, and dried in an oven at 60 °C for 4 h.

Preparation of CuO NW/CF: Cu(OH)_2_ NW/CF prepared in the previous step was used in a muffer furnace, starting at 20°C, reaching 200°C through 90 min, then kept for 120 min, and then dropped to 20°C through 90 min, and named CuO NW/CF.

Preparation of acid group anion (AGA)-CoNi LDH/CuO NW/CF: The AGA-CoNi LDH/CuO NW/CF was synthesized as follows: 2 mmol of nickel nitrate, 1.5 mmol of cobalt nitrate and 2 g of urea were dissolved into 70 mL of deionized water. The mixture was then added to a 100 mL Teflon-lined stainless-steel hydrothermal reactor, along with a piece of CuO NW/CF at an angle of 60 degree against the inner wall of Teflon-lining, then put into an electric oven heated at 110^o^C for 6 h. After the hydrothermal reaction, the NO_3_-CoNi LDH/CuO NW/CF was rinsed with deionized water and dried in an oven at 60°C for 4 h. Then, cobalt nitrate and nickel nitrate were replaced with cobalt chloride and nickel chloride to prepare Cl-CoNi LDH/CuO NW/CF. Cobalt nitrate and nickel nitrate were replaced with cobalt sulfate and nickel sulfate to prepare SO_4_-CoNi LDH/CuO NW/CF. 1 mmol of sodium molybdate or sodium tungstate was added to the mixed solution to prepare MoO_4_-CoNi LDH/CuO NW/CF or WO_4_-CoNi LDH/CuO NW/CF.

- 1. **Characterization**

The morphology and microstructure of the as-prepared samples were investigated by scanning electron microscope (SEM Zeiss Gemini SEM 500) and Lorenz Transmission Electron Microscope (TEM, Talos F200X). The X-ray diffraction (XRD) patterns of samples were recorded on a Bruker D8 ADVANCE using a Cu Kα radiation (*λ*=1.5418 Å). The Raman measurement (LabRAM HR Evolution, with an excitation of 785 nm laser light) was used to analyze the composition of samples. The X-ray photoelectron spectroscopy (XPS) spectrums of samples were obtained from a Thermo Scientific EscaLab 250Xi with an Al monochromatic X-ray source (1486.6 eV). All binding energies (BEs) were referenced to the C 1s hydrocarbon peak at 284.8 eV.

- 1. **In-situ Raman spectroscopy.**

The in-situ Raman measurements were performed on a confocal Raman specctrometer (InVia Qontor) using an excitation of 785 nm laser, the potential was controlled by an electrochemical workstation (CHI 660E, Shanghai CHENHUA Instrument Co., LTD). In-situ electrochemical Raman experiment was employed in a H-type Raman cell separated by a Nafion proton exchange membrane (Dupont Nafion 117). MoO_4_-CoNi LDH/CuO NW/CF was placed flat on the bottom of the Raman in-situ pool as a working electrode to keep the plane of the sample perpendicular to the incident laser. A platinum wire and an Hg/HgO electrode (filled with 1 M KOH solution) were used as counter and reference electrodes, respectively. A mixed solution (1M KOH+0.05 M NaNO_3_) was used as the electrolyte.

- 1. **Ex-situ X-ray diffraction.**

Ex-situ XRD measurements were conducted using Bruker D8 ADVANCE XRD spectrometers equipped with a Cu Ka X-ray source. The prepared electrodes underwent electrochemical pretreatment in an H-type electrolytic cell under NitRR for approximately 0.5 hours, while the electrolyzer was purged with an inert gas. Subsequently, the electrodes were transferred to the XRD analysis chamber via a vacuum bag. Throughout the transfer process, samples were prepared and kept under vacuum conditions to prevent exposure to air.

- 1. **In situ EIS Experiments**

In an electrolyte solution saturated with Ar gas, in-situ electrochemical impedance spectroscopy (EIS) measurements were conducted using an electrochemical workstation equipped with a three-electrode system (working electrode, reference electrode, and platinum counter electrode) at different applied potentials. After stabilizing the working electrode at the open-circuit potential, a constant DC voltage was applied, followed by a 5-minute equilibration period to ensure steady-state conditions. EIS spectra were obtained in the frequency range of 10^5^ Hz to 10^-2^ Hz with an AC perturbation amplitude of 5 mV, while maintaining the applied DC bias. This process was gradually repeated within the target voltage range of 0.2 *vs.* RHE to -0.3 *vs.* RHE. The Nyquist plots obtained were analyzed through equivalent circuit modeling to extract parameters such as charge transfer resistance (R_ct_) and double-layer capacitance, thereby enabling the assessment of voltage-dependent interfacial dynamics and surface processes.

- 1. **CV Experiments**

Cyclic voltammetry (CV) tests were conducted at different initial potentials using a three-electrode system (working electrode, reference electrode, and platinum counter electrode) in degassed electrolyte. After stabilizing the open-circuit potential, the potential window was adjusted successively by gradually changing the initial scan voltage, while maintaining a constant scan rate and upper limit potential. The current responses in multiple cycles were recorded to evaluate reproducibility, and the peak potential shifts or current density changes were analyzed to assess the voltage-dependent redox behavior, surface reactivity, and kinetic limitations of the electrode material.

- 1. **Electron paramagnetic resonance (EPR)** **Experiments**

5,5-dimethyl-1-pyrroline N-oxide (DMPO) was used to capture the instable hydrogen radical to form the DMPO-H adduct to generate EPR spectra. In the experiments, 0.1 mL electrolyte was mixed with 100 μL DMPO and was deoxygenated by bubbling Ar. The constant current electrolysis was carried out for 10 min in the H-type cell under the protection of Ar.

- 1. **Electrochemical measurement**

The electrochemical tests were performed using a three-electrode system connected to the CHI660D electrochemical workstation in a typical H-type cell. The H-type cell was separated by a Nafion 117 membrane (Dupont) that was pretreated following reported procedures. The catalysts were used as the working electrode, while Hg/HgO (filled with 1 M KOH solution) and platinum mesh were used as the reference and counter electrodes, respectively. The electrolytes were Ar-saturated 1 M KOH (pH=14) containing different concentrations of NO_3_^−^. All potentials were calibrated to the RHE reference scale using *E*_RHE_ = *E*_HgO/Hg_+0.098+0.0591×pH. The current density was normalized to the geometric electrode area (~2 cm^2^). Note that the electrode area was 2 cm^2^ for tests in 0.05 M nitrate. NitRR activity was determined by linear sweep voltammetry (LSV) with a scan rate of 5 mV/s. The stabilization experiments of MoO_4_-CoNi LDH/CuO NW/CF at voltages of −0.2 V vs. RHE were by the Amperometric *i-t* Curve (*i-t*) methods, respectively. Electrochemical impedance spectroscopy (EIS) measurements were performed over a frequency range of 0.1-1.0 × 106 Hz by applying an AC amplitude of 50 mV.

- 1. **UV-vis analysis**

The concentrations of electrolyte ions before and after the test were measured by UV-vis spectrophotometer. To ensure accurate measurements, electrolytes were neutralized and diluted appropriately to match the range of the calibration curve as follows:

1.5.1 Determination of nitrate-N

**NO_3_^−^ quantification**. After neutralization and dilution, 1 mL of post-electrolysis electrolytes was diluted to 4 mL and 1 mL of 1 M HCl and 0.1 mL of a sulfamic acid solution were added. The mixed solution was shaken to obtain a homogeneous solution. A UV-Vis spectrophotometer was used to record the absorption intensities at wavelengths of 220 nm and 275 nm. The calculated absorbance value A (A = A_220nm_ – 2×A_275nm_) was linearly correlated with the NO_3_^−^ concentrations. To quantify the amount of NO_3_^−^, a calibration curve was obtained using a NaNO_3_ standard solution. Prior to the calibration, sodium nitrate crystals are dried to constant weight at 105-110 ℃.

**NO_2_^−^ quantification.** A mixture of p-aminobenzenesulfonamide (4 g), N-(1-Naphthyl) ethylenediamine dihydrochloride (0.2 g), ultrapure water (50 mL), and phosphoric acid (10 mL, density=1.70 g/mL) was obtained to prepare the color reagent. Subsequently, after neutralization, 1 mL of the electrolyte sample was combined with 1 mL of the color reagent and diluted to a total volume of 50 mL in a colorimetric tube to match the detection range. For measurement purposes, approximately 5 mL of the uniformly mixed solution was placed in a 1 cm cuvette, while another 5 mL of deionized water served as the reference solution in a separate 1 cm cuvette. The absorption of NO_2_^-^ at a wavelength of 540 nm was then recorded. To establish a concentration-absorbance curve, a series of standard sodium nitrite solutions were employed for calibration. Prior to the calibration, sodium nitrite crystals are dried to constant weight at 105-110 ℃.

**NH_4_^+^ quantification.** To detect NH_4_^+^ ions, Nessler's reagent was utilized as the color reagent. The detection process for NH_4_^+^ is as follows: 1 mL of the electrolyte sample to be analyzed is extracted from the electrolytic cell, neutralized and diluted, and transferred to the colorimetric tube. Next, 1 mL of potassium sodium tartrate solution (density=0.5 g/mL) was added to the sample and thoroughly mixed. 1 mL of Nessler's reagent was added to the mixture, and the resulting solution was then diluted to a total volume of 50 mL to match the detection range. For measurement purposes, approximately 5 mL of the uniformly mixed solution was placed in a 1 cm cuvette, while another 5 mL of deionized water served as the reference solution in a separate 1 cm cuvette. The absorption of NH_4_^+^ at a wavelength of 420 nm was then recorded. To establish a concentration-absorbance curve, a series of standard ammonium chloride solutions were employed for calibration. Prior to the calibration, ammonium chloride crystals are dried to constant weight at 105-110 ℃.

- 1. **Computational Formula in the three-electrode system.**

1. For the NitRR reaction, the yield rate of products can be calculated as the following equation:

*ν* = (*c* × *V*) / (*t* × *S*) (mmol h^−1^ cm^−2^)

where *c* (mmol L^−1^) is the measured concentration of NO_2_^−^ or NH_3_, *V* (L) is the volume of electrolyte, *t* (h) is the time of chronoamperometry testing, and *S* is the effective geometric area of the Cu-based electrode.

1. The Faradaic efficiency (FE) was calculated by using the following equation:

FE = (n*F* × *c* ×*V*)/(*M* × *Q*)

where n is the number of electron transfer *F* (C·mol^−1^) is the Faraday constant, *M* (g·mol^−1^) is the molecular mass of ammonia or nitrite, *Q* (C)is the amount of total charge of electrolysis by the integration of chronoamperometry curves.

1. NH_3_ selectivity in this work was defined and calculated according to the following equation:

Selectivity(NH_3_)=[*c*(NH_3_)]/([*c*(NO_2_^−^)]+ *c*(NH_3_))

where *C*(NH_3_) and *C*(NO_2_^−^) (mmol L^−1^) are the concentrations of NH_3_ and NO_2_^−^ after electrolysis, respectively.

1. Kinetics of tandem reactions.

Nitrate/nitrite reduction reactions were conducted under ambient temperature and pressure conditions. The reaction rate (*k* with unit of h^−1^) was calculated by assuming first-order dependence on nitrate/nitrite concentration:^1, 2^

The reaction series are divided into NO_3_^−^ being reduced to NO_2_^−^, and then NO_2_^−^ being further reduced to NH_4_^+^.

$$\text{NO}_{\text{3}}^{\text{-}} \underset{\to}{k_{1}} \text{NO}_{\text{2}}^{\text{-}} \underset{\to}{k_{1}} \mathrm{NH}_{4}^{+}$$

*k*_1_ is the rate constant for the reduction of NO_3_^−^ to NO_2_^−^; *k*_2_ is the rate constant for the reduction of NO_2_^−^ to NH_4_^+^.

Assuming the reduction of NO_3_^−^ to NO_2_^−^ and the reduction of NO_2_^−^ to NH_4_^+^ are first-order reactions, the differential equations for the concentration changes of NO_3_^−^, NO_2_^−^, and NH_4_^+^ over time can be written as follows:^3, 4, 5, 6, 7^.

$$\frac{d\text{c(NO}_{\text{3}}^{\text{-}})}{d\text{t}} = -k_{1}\text{c(NO}_{\text{3}}^{\text{-}})$$

$$\frac{d\text{c(NO}_{\text{2}}^{\text{-}})}{d\text{t}} = k_{1}\text{c(NO}_{\text{3}}^{\text{-}})\text{ }- k_{2}\text{c(NO}_{\text{2}}^{\text{-}})$$

$$\frac{d\text{c(NH}_{\text{4}}^{\text{+}})}{d\text{t}} = k_{2}\text{c(NO}_{\text{2}}^{\text{-}})$$

The initial concentration of NO_3_^−^ is c(NO_3_^−^)_0_, and the initial concentrations of NO_2_^−^ and NH_4_^+^ are 0. The change in concentration of NO_3_^−^ over time is obtained by separating variables in the equation and then integrating both sides.

$$\frac{d\text{c(NO}_{\text{3}}^{\text{-}})}{\text{c(NO}_{\text{3}}^{\text{-}})} = -k_{1}d\text{t}$$

$$\int_{{{c(NO}_{3}^{-})}_{0}}^{{{c(NO}_{3}^{-})}_{t}} \frac{d\text{c(NO}_{\text{3}}^{\text{-}})}{\text{c(NO}_{\text{3}}^{\text{-}})} = \int_{0}^{t} -k_{1}d\text{t}$$

$$\text{c(NO}_{\text{3}}^{\text{-}}) = {{c(NO}_{3}^{-})}_{0}{\times e}^{-k_{1}t}$$

Translate the above results into the following formula.

$$\frac{d\text{c(NO}_{\text{2}}^{\text{-}})}{d\text{t}} = k_{1}\text{×c(NO}_{\text{3}}^{\text{-}})\text{ }- k_{2}\text{×c(NO}_{\text{2}}^{\text{-}})$$

$$\frac{d\text{c(NO}_{\text{2}}^{\text{-}})}{d\text{t}} + k_{2}\text{c(NO}_{\text{2}}^{\text{-}})= {{c(NO}_{3}^{-})}_{0}{\times k}_{1}{\times e}^{-k_{1}t}$$

$$(\frac{d\text{c(NO}_{\text{2}}^{\text{-}})}{d\text{t}} + k_{2}\times\text{c(NO}_{\text{2}}^{\text{-}}))\times e^{k_{2}\text{t}}= {{c(NO}_{3}^{-})}_{0}{\times k}_{1}{\times e}^{-k_{1}t}\times e^{k_{2}\text{t}}$$

$$\frac{d}{d\text{t}} \text{(c(NO}_{\text{2}}^{\text{-}}))\times e^{k_{2}\text{t}})= {{c(NO}_{3}^{-})}_{0}{\times k}_{1}{\times e}^{-{(k}_{1}-k_{2})t}$$

$$\int_{{{c(NO}_{2}^{-})}_{0}=0}^{{{c(NO}_{2}^{-})}_{t}} \text{d(c(NO}_{\text{2}}^{\text{-}}))\times e^{k_{2}\text{t}}) = \int_{0}^{t} {{c(NO}_{3}^{-})}_{0}{\times k}_{1}{\times e}^{-{(k}_{1}-k_{2})t}dt$$

$$\text{c(NO}_{\text{2}}^{\text{-}})\times e^{k_{2}\text{t}}= -\frac{{{c(NO}_{3}^{-})}_{0}{\times k}_{1}}{k_{1}-k_{2}}(e^{-{(k}_{1}-k_{2})t}-1)$$

$$\text{c(NO}_{\text{2}}^{\text{-}})= \frac{{{c(NO}_{3}^{-})}_{0}{\times k}_{1}}{k_{1}-k_{2}}(e^{{-k}_{2}t}-e^{{-k}_{1}t})$$

Translate the above results into the following formula.

$$\frac{d\text{c(NH}_{\text{4}}^{\text{+}})}{d\text{t}} = k_{2}\text{c(NO}_{\text{2}}^{\text{-}})$$

$$\frac{d\text{c(NH}_{\text{4}}^{\text{+}})}{d\text{t}} = \frac{{{c(NO}_{3}^{-})}_{0}{\times k}_{1}\times k_{2}}{k_{1}-k_{2}}(e^{{-k}_{2}t}-e^{{-k}_{1}t})$$

$$\int_{0}^{\text{c(NH}_{\text{4}}^{\text{+}})} d\text{c(NH}_{\text{4}}^{\text{+}}) = \frac{{{c(NO}_{3}^{-})}_{0}{\times k}_{1}\times k_{2}}{k_{1}-k_{2}}\int_{0}^{t} (e^{{-k}_{2}t}-e^{{-k}_{1}t})dt$$

$$\text{c(NH}_{\text{4}}^{\text{+}}) = \frac{{{c(NO}_{3}^{-})}_{0}{\times k}_{1}\times k_{2}}{k_{1}-k_{2}}(-\frac{1}{k_{2}}\left( e^{{-k}_{2}t}-1 \right)+\frac{1}{k_{1}}(e^{{-k}_{1}t}-1))$$

$$\text{c(NH}_{\text{4}}^{\text{+}}) = {{c(NO}_{3}^{-})}_{0}\times(1+\frac{k_{1}{\times e}^{{-k}_{2}t}-k_{2}e^{{-k}_{1}t}}{k_{2}-k_{1}})$$

The relationship between *k*_1_ and *k*_2_ and the amount of charge *Q*.

$$\text{k}_{1} = a_{1}\times Q+b_{1}$$

$$k_{\text{2}}= a_{2}\times e^{b_{2}\times Q}+c_{2}$$

- 1. **Computational methods**

The Vienna Ab Initio Package (VASP) was employed to perform all the density DFT calculations within the generalized gradient approximation (GGA) using the Perdew-Burke-Ernzerhof (PBE) formulation.^8, 9^ The projected augmented wave (PAW) potentials were chosen to describe the ionic cores and took valence electrons into account using a plane-wave basis set with a kinetic energy cutoff of 450 eV.^10^ The Brillouin zone was built with a (4 × 4 × 1) gamma centered k-point mesh for all models in the optimization of the supercell structure. The force residue for relaxation of all the atoms was set as 0.02 eV·Å^−1^. The Cu_2_O (1 1 1) and CuO (0 0 2) surfaces are covered by Cu atoms, thereby simulating the reduction of Cu_2_O (1 1 1) and CuO (0 0 2) regions. A vacuum space of 15Å is set along the z direction to ensure no interaction between adjacent images. Subsequently, intermediate molecules are loaded onto the surface. In the case of Cu/Cu_2_O (1 1 1), the bottom atomic layer remains fixed while the top three atomic layers undergo sufficient relaxation. For Cu/CuO (0 0 2), all atoms experience adequate relaxation.

The adsorption energy of NO_3_^−^ on the surface of the catalyst was calculated by:

∆E_A_ = *E**_A_ – *E** – *E*_A_

where *E**_A_, *E** and *E*_A_ denote the energy of adsorbed system, clear surface, and a single NO_3_^−^ group, respectively.^11^

According to the method presented by Nørskov, the Gibbs free energy diagrams were estimated by the following equation:^12^

∆*G* = ∆E + ∆*ZPE* – *T*∆*S* + Δ*G*_U_

where ∆E, and Δ*ZPE* denote the calculated total energy and zero-point energy, respectively. Δ*G*_U_ = –eU (U: potential); *T* and ∆*S* denote temperature and entropy change, respectively. Here, *T* = 300 K was considered.

- 1. **Zn-Nitrate Batter**

The schematic diagram of an aqueous rechargeable zinc nitrate (Zn-NO_3_) battery is depicted in Figure 5a. In the anode, a mixed solution comprising 3 mol·L^−1^ KOH and 0.02 mol·L^−1^ Zn(CH_3_COO)_2_ is employed, while the cathode solution consists of 3 mol·L^−1^ KOH and 0.5 mol·L^−1^ NaNO_3_. Nafion 117 membranes are utilized for efficient electrolyte separation. During discharge, the dissolution of Zn metal on the anode liberates electrons, facilitating NitRR on the cathode via electron transport. Upon charging, water undergoes oxidation to generate O_2_, while Zn(OH)_4_^2−^ formation occurs at the anode to yield Zn, thereby initiating subsequent cell reactions.

Discharge reaction:

4Zn+NO_3_^−^+7H_2_O+6OH^−^→4Zn(OH)_4_^2−^+NH_4_^+^, *E*_discharge_ = 1.152 V

Charge reaction:

2Zn(OH)_4_^2−^→2Zn+2H_2_O+4OH^−^+O_2_, *E*_charge_ = 1.650 V

Overall reaction:

NO_3_^−^+3H_2_O→NH_4_^+^+2OH^−^+2O_2_

**2 Supporting Figures**


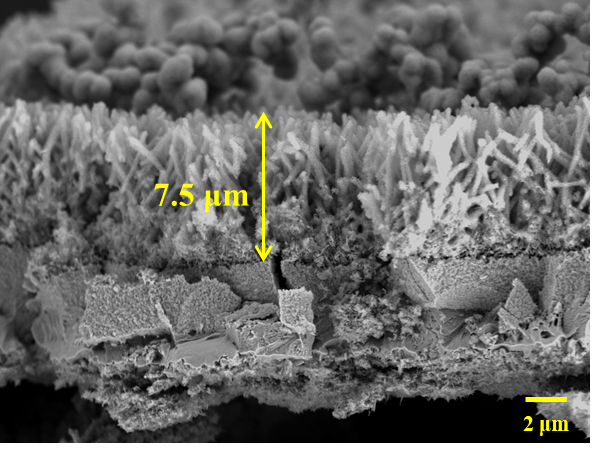


Figure S1 Cross-section SEM image of the MoO_4_-CoNi LDH/CuO NW/CF composite electrode.


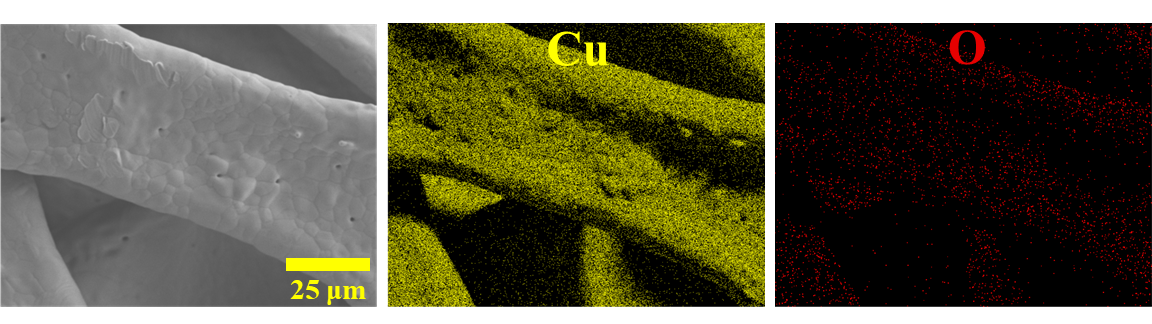


Figure S2 SEM image and elemental mapping of the CF.


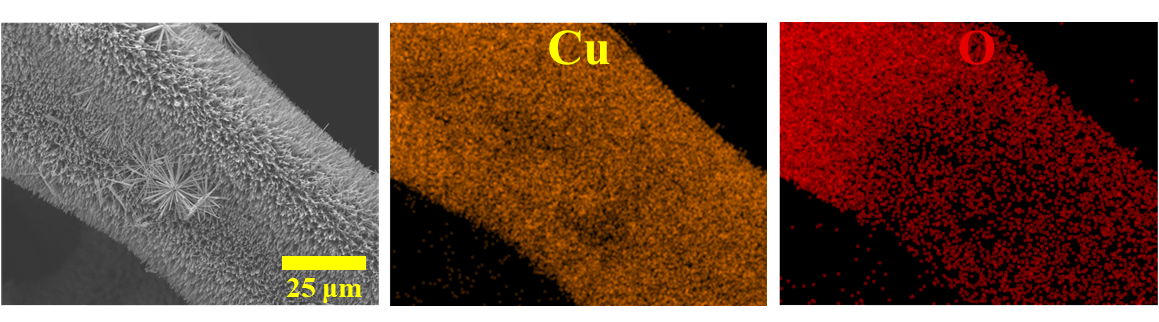


Figure S3 SEM image and elemental mapping of the Cu(OH)_2_ NW/CF.


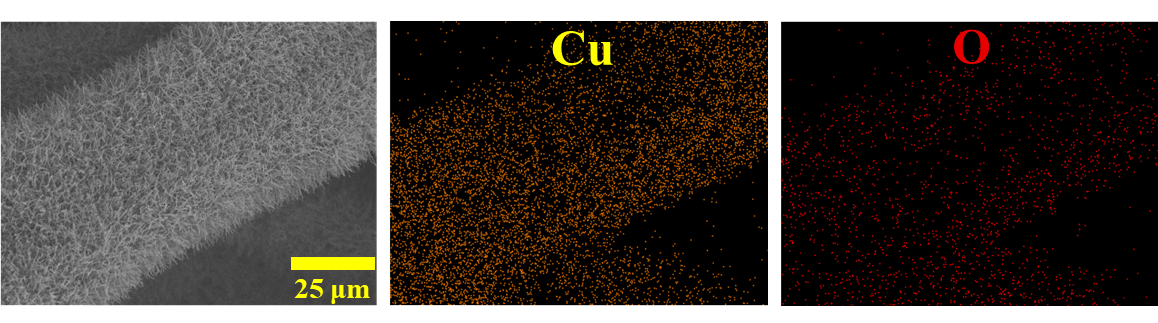


Figure S4 SEM image and elemental mapping of the CuO NW/CF.


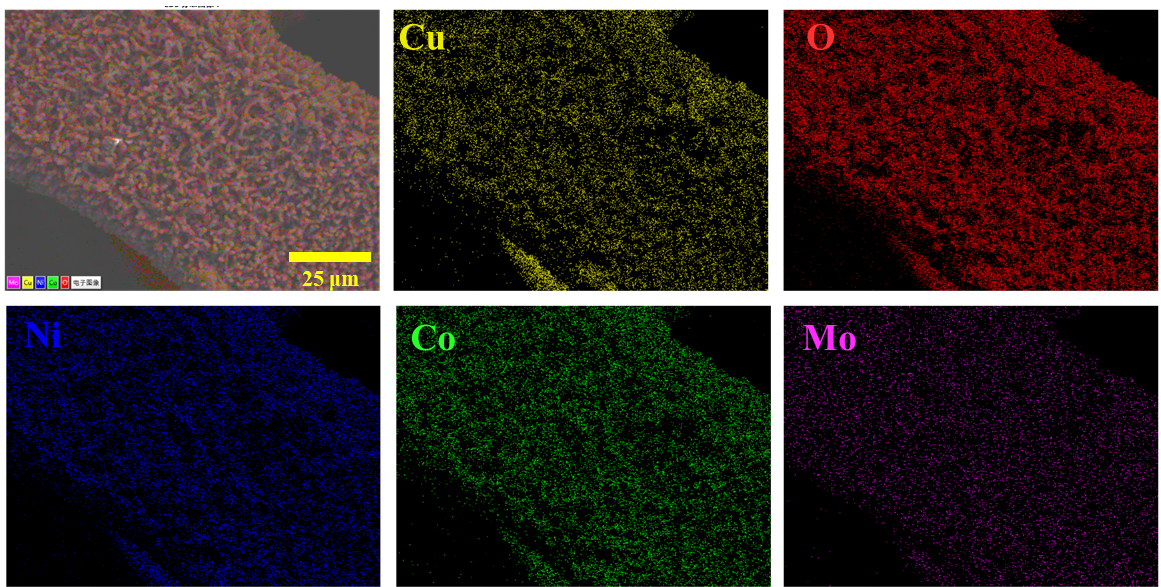


Figure S5 SEM image and elemental mapping of the MoO_4_-CoNi LDH/CuO NW/CF.


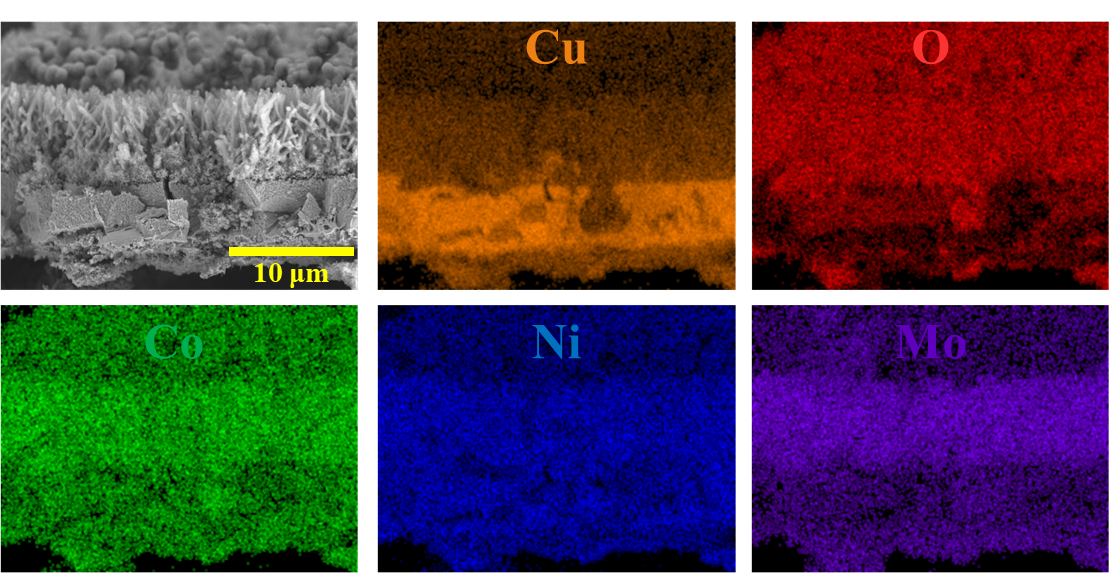


Figure S6 Cross-section SEM images and elemental mapping of the MoO_4_-CoNi LDH/CuO NW/CF.


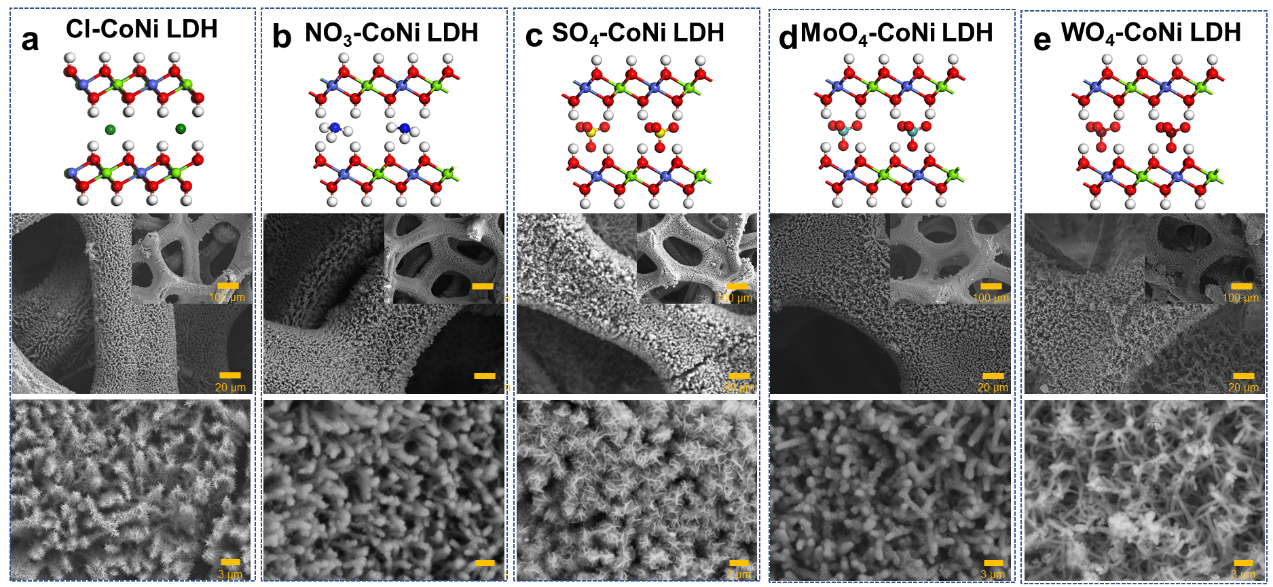


Figure S7 SEM images of the NO_3_-NiCo LDH/CuO NW/CF (a), Cl-NiCo LDH/CuO NW/CF (b), SO_4_-NiCo LDH/CuO NW/CF (c), MoO_4_-NiCo LDH/CuO NW/CF (d) and WO_4_-NiCo LDH/CuO NW/CF (e).


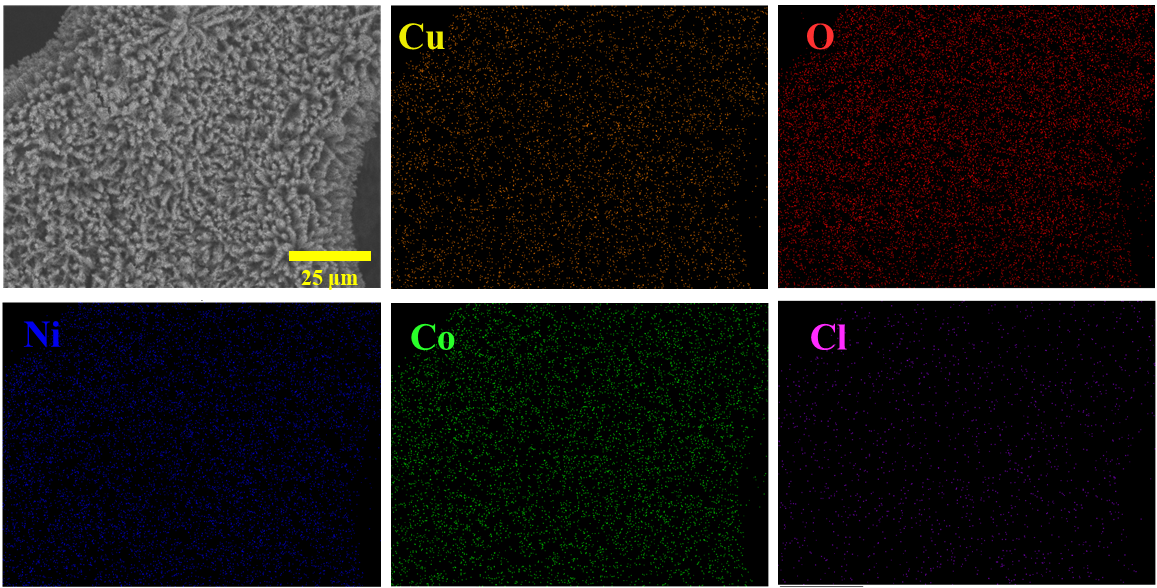


Figure S8 SEM image and elemental mapping of the Cl-CoNi LDH/CuO NW/CF.


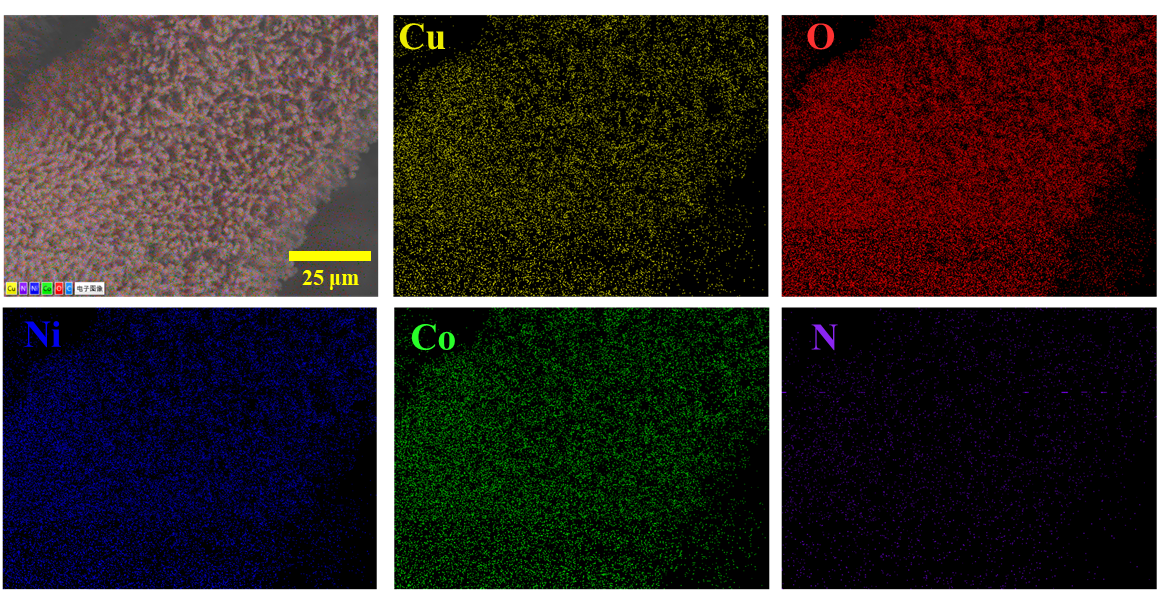


Figure S9 SEM image and elemental mapping of the NO_3_-CoNi LDH/CuO NW/CF.


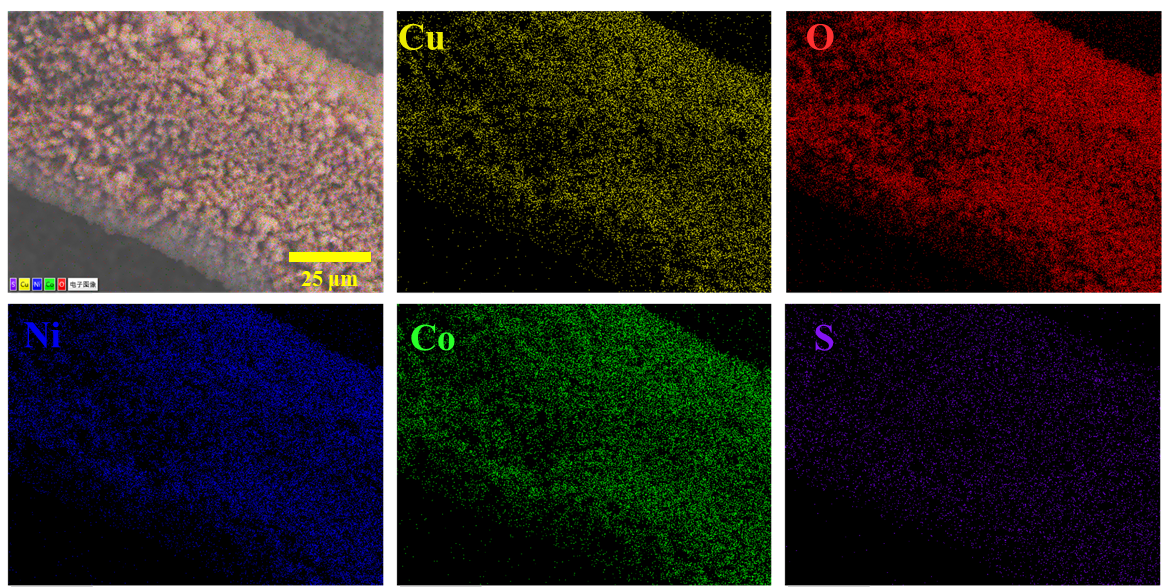


Figure S10 SEM image and elemental mapping of the SO_4_-CoNi LDH/CuO NW/CF.


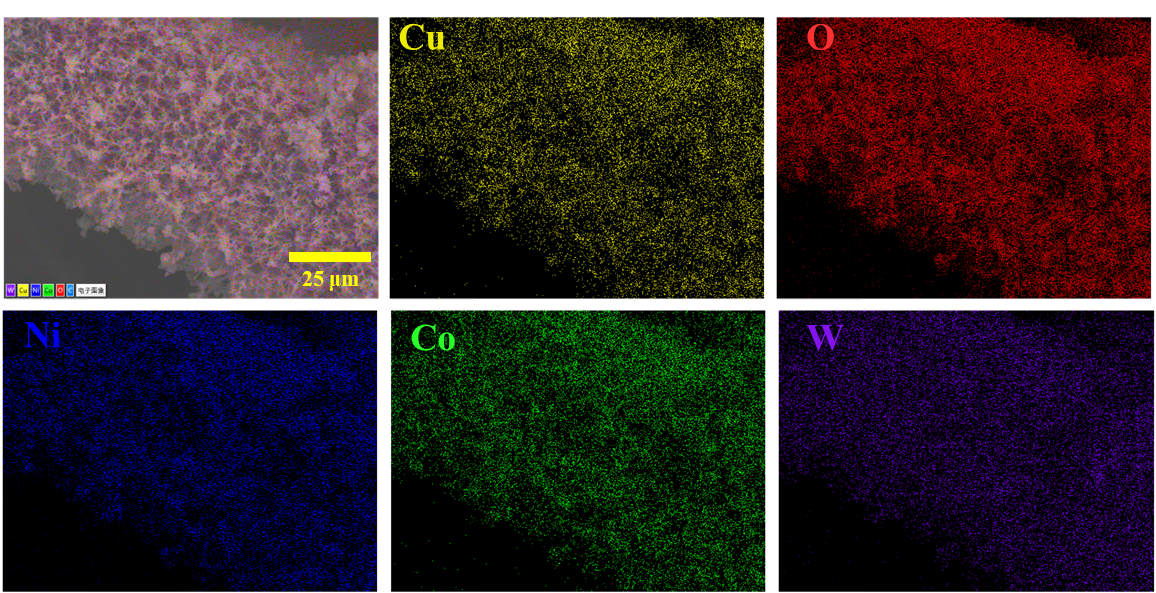


Figure S11 SEM image and elemental mapping of the WO_4_-CoNi LDH/CuO NW/CF.


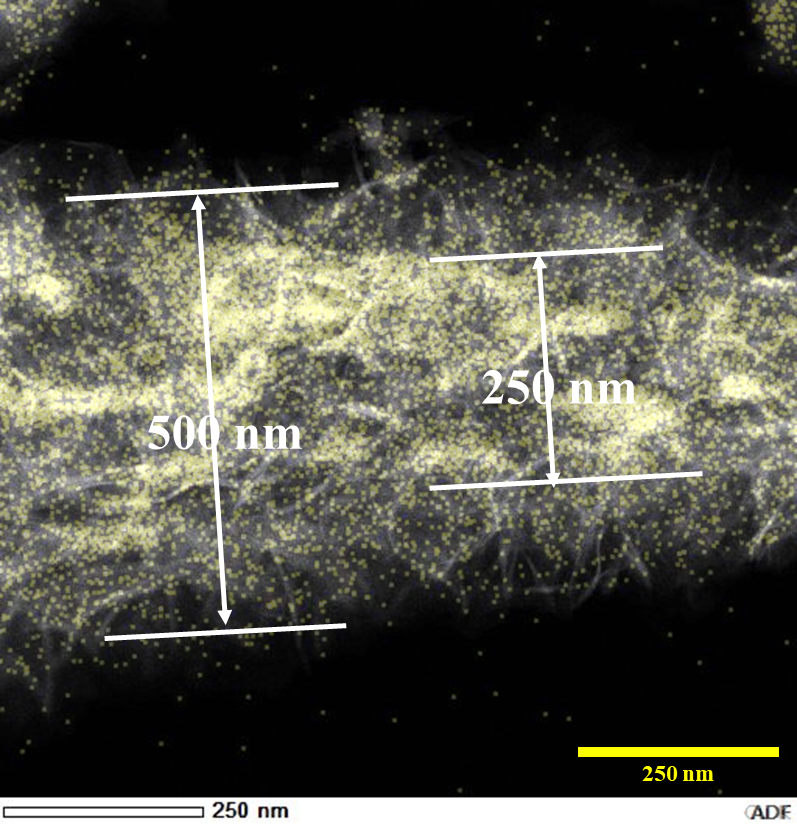


Figure S12 Typical TEM element mapping image of MoO_4_-CoNi LDH/CuO NW/CF


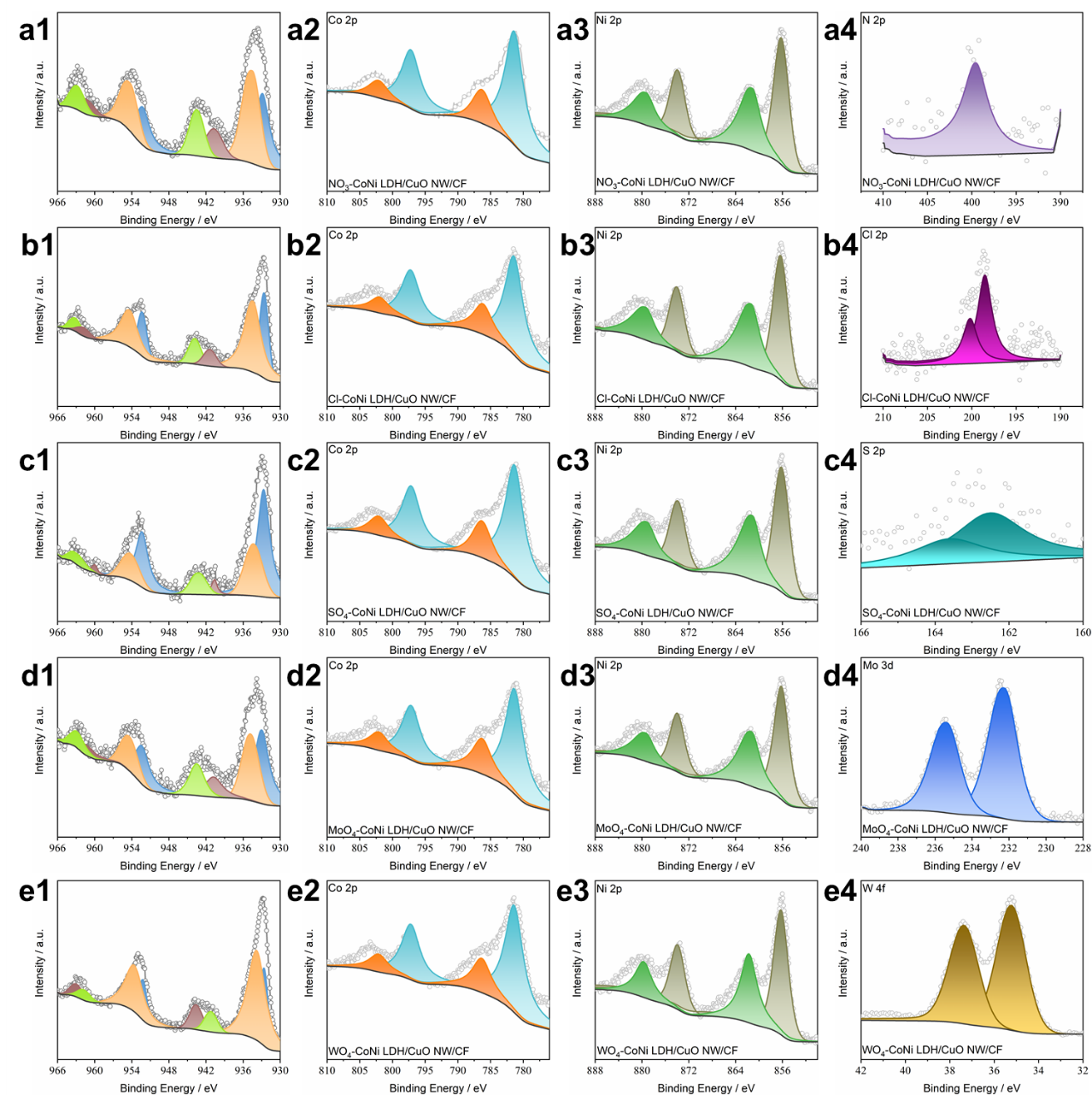


Figure S13 XPS patterns of (a) NO_3_-NiCo LDH/CuO NW/CF, (b) Cl-NiCo LDH/CuO NW/CF, (c) SO_4_-NiCo LDH/CuO NW/CF, (d) MoO_4_-NiCo LDH/CuO NW/CF and (e) WO_4_-NiCo LDH/CuO NW/CF.


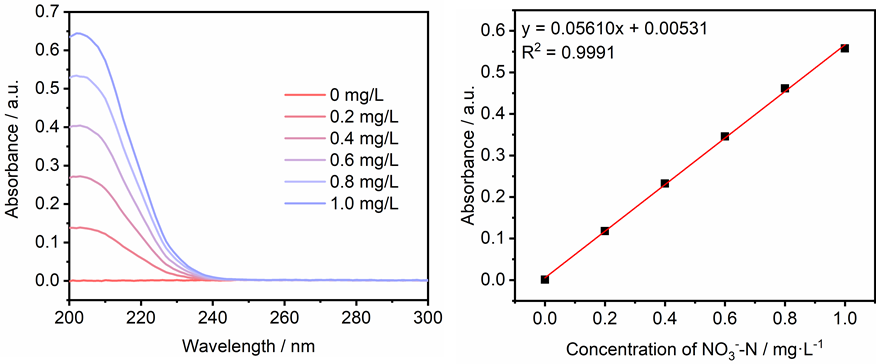


Figure S14 NO_3_^-^ calibration curve with different concentration of NaNO_3_ solutions using UV-vis absorption spectroscopy. (a) UV-vis adsorption spectra of NO_3_^-^ ions and (b) the corresponding linear fitting results of the calibration curve.


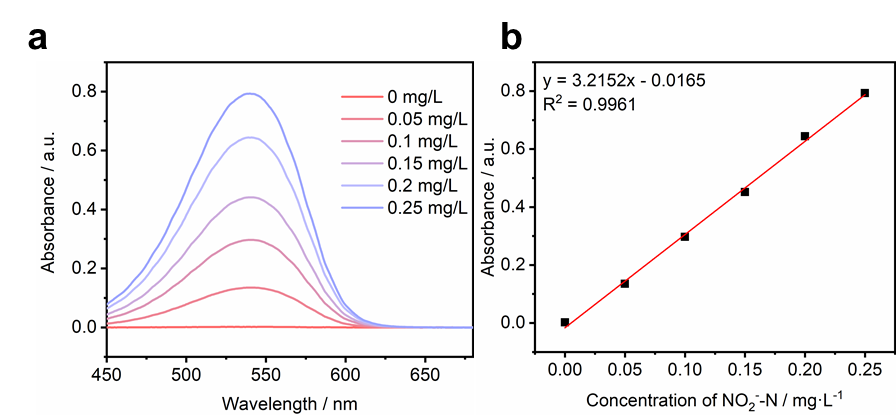


Figure S15 NO_2_^-^ calibration curve with different concentration of NaNO_2_ solutions using UV-vis absorption spectroscopy. (a) UV-vis adsorption spectra of NO_2_^-^ ions and (b) the corresponding linear fitting results of the calibration curve.


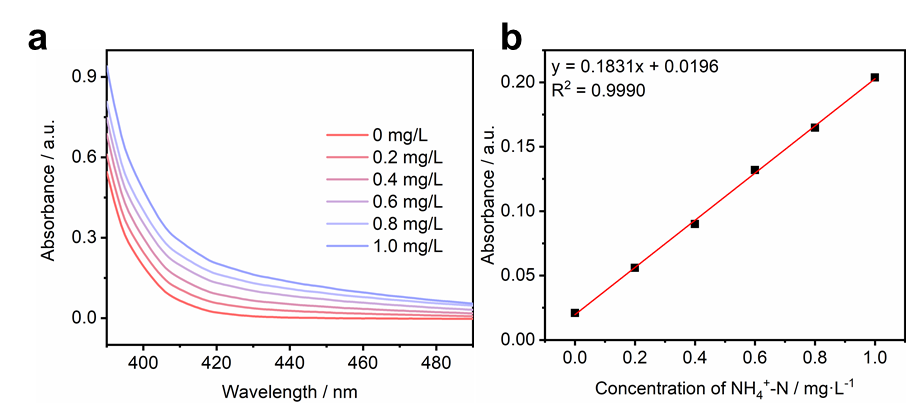


Figure S16 NH_4_^+^ calibration curve with different concentration of NH_4_Cl solutions using UV-vis absorption spectroscopy. (a) UV-vis adsorption spectra of NH_4_^+^ ions and b) the corresponding linear fitting results of the calibration curve.





Figure S17 The NH_3_ Faraday efficiency and yield of CoNi LDH electrodes with different ionic intercalation under -0.2 V condition





Figure S18 The corresponding Faraday efficiencies (FE) of NO_2_^−^ for CuO NW/CF, NO_3_-CoNi LDH/CuO NW/CF and MoO_4_-CoNi LDH/CuO NW/CF in a 1 mol·L^−1^ KOH with 0.05 mol·L^−1^ NO_3_^−^ electrolyte at different potentials.





Figure S19 NH_3_ and NO_2_^−^ selectivity of MoO_4_-CoNi LDH/CuO NW/CF


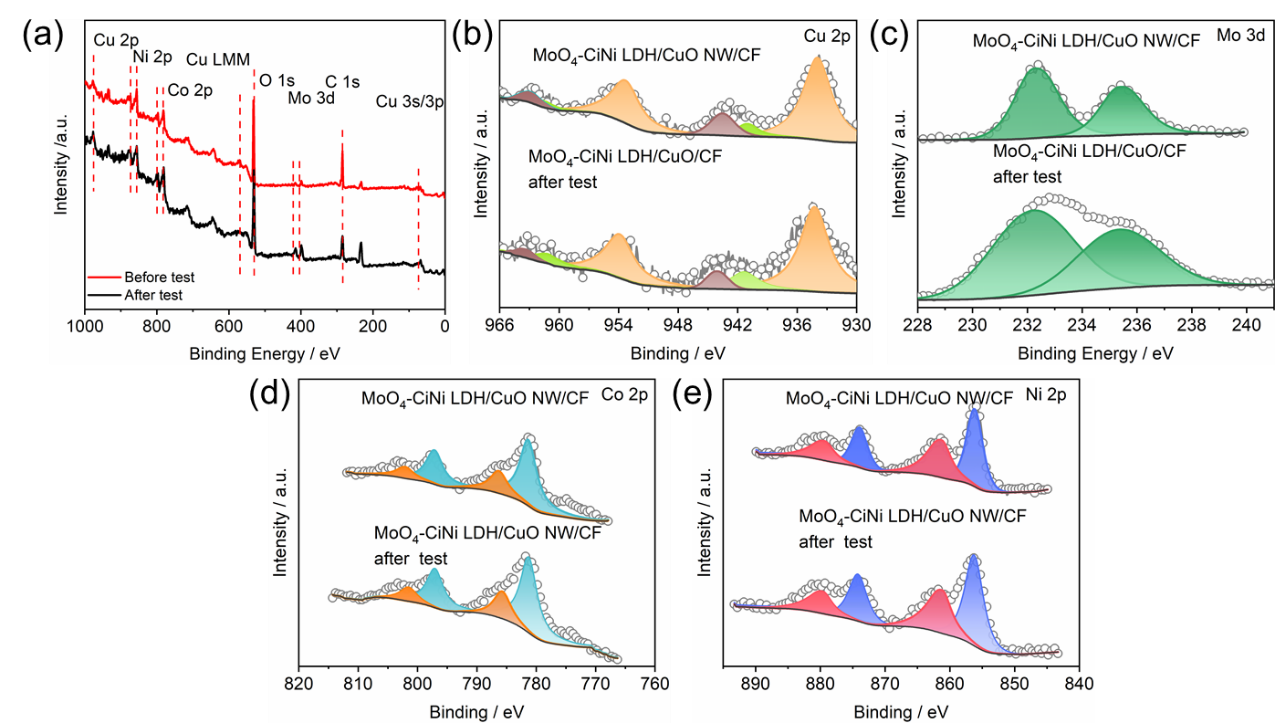


Figure S20 The full scan (a), Cu 2p (b), Mo 3d (c) ,Co 2p and Ni 2p (d) XPS spectra of MoO_4_-CoNi LDH/CuO NW/CF and MoO_4_-CoNi LDH /CuO NW/CF electrodes after the test.





Figure S21 The concentrations of metal ions after different cycles





Figure S22 The reaction kinetics of MoO_4_-CoNi LDH/CuO NW/CF fitting at −0.2 V vs. RHE.


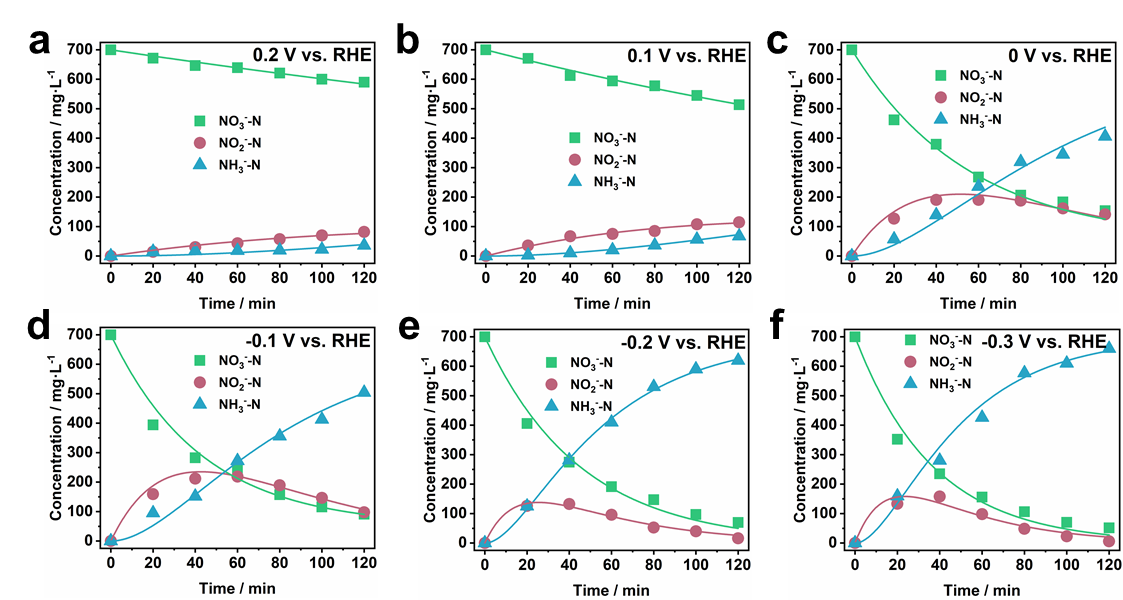


Figure S23 The reaction kinetics of MoO_4_-CoNi LDH/CuO NW/CF fitting at different voltages (0.2 - −0.3 V vs. RHE).





Figure S24 Variation in concentration of NO_2_^−^ in the electrolyte after the redox reaction with CuO NW/CF for different times, without an applied potential.


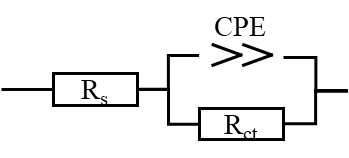


Figure S25 The equivalent circuit for modeling the measured electrochemical response of MoO_4_-CoNi LDH. *R*_ct_ represents charge transfer resistance, *R*_s_ represents solution resistance and CPE_ct_ is related to double layered capacitance.


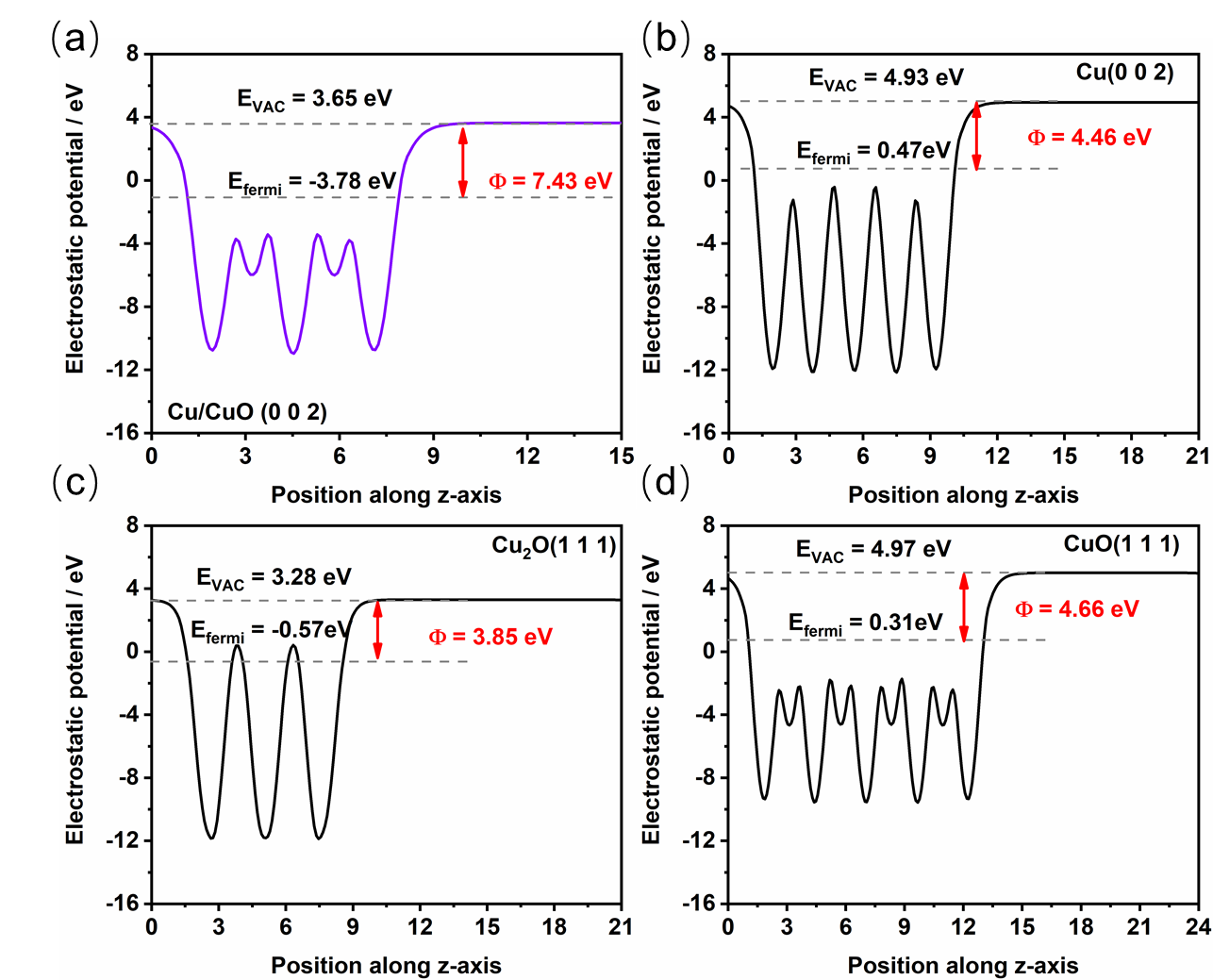


Figure S26 Electrostatic potentials of Cu/CuO (0 0 2), Cu(002), Cu_2_O(1 1 1) and CuO(1 1 1) surface.


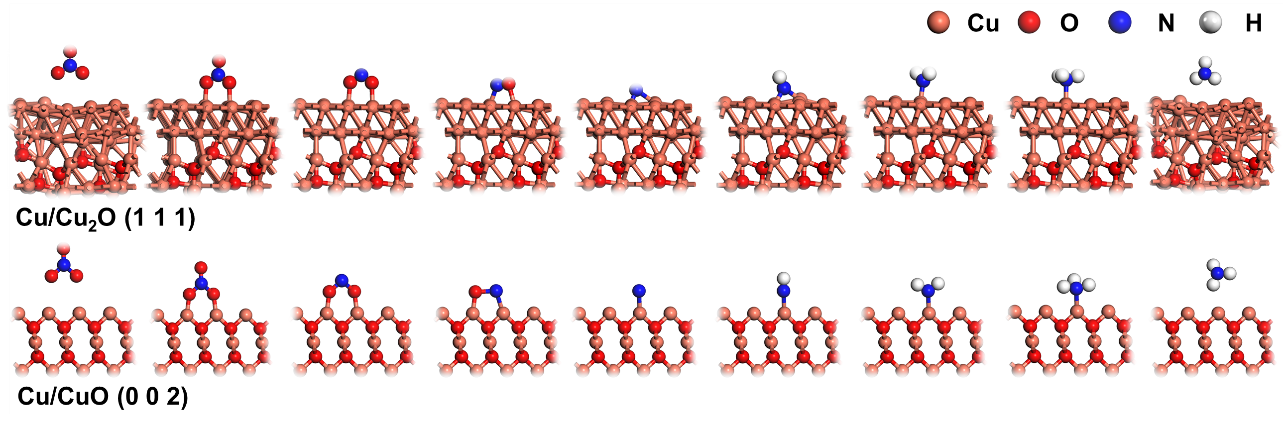


Figure S27 The related intermediates during the reduction from NO_3_^−^ to NH_3_ by Cu/Cu_2_O (1 1 1) and Cu/CuO (0 0 2).


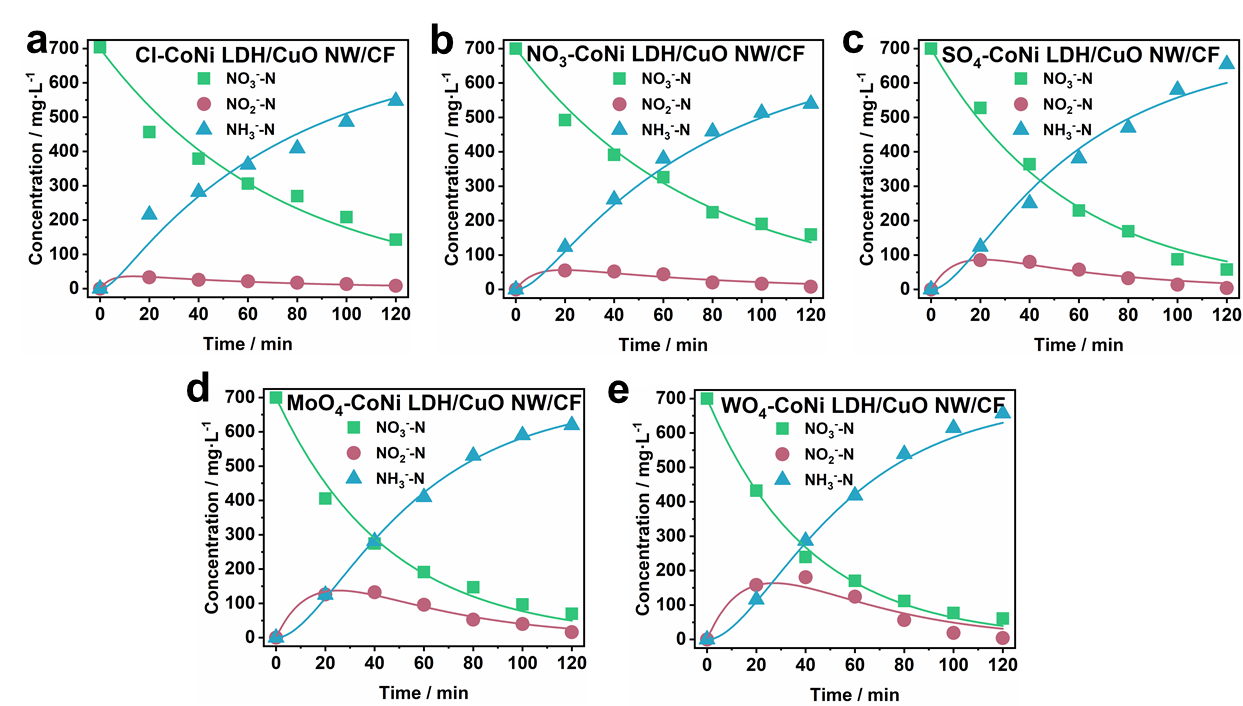


Figure S28 Reaction kinetics of CoNi LDH/CuO NW/CF series under different anion intercalation (Cl^−^, NO_3_^−^, SO_4_^2−^, MoO_4_^2−^ and WO_4_^2−^).





Figure S29 3D colormap surface plot and corresponding colortour maps for of NO_2_^−^ concentration on the rate constant of nitrite reduction by different nitrates (*k*_1_) and the rate constant of nitrite reduction to ammonia (*k*_2_).





Figure S30 Effects of starting potential on CV curves of CuO NW/CF in Ar-saturated 1 M KOH solution.





Figure S31 Under TBA masking condition, the LSV of MoO_4_-CoNi LDH/CuO NW/CF electrode


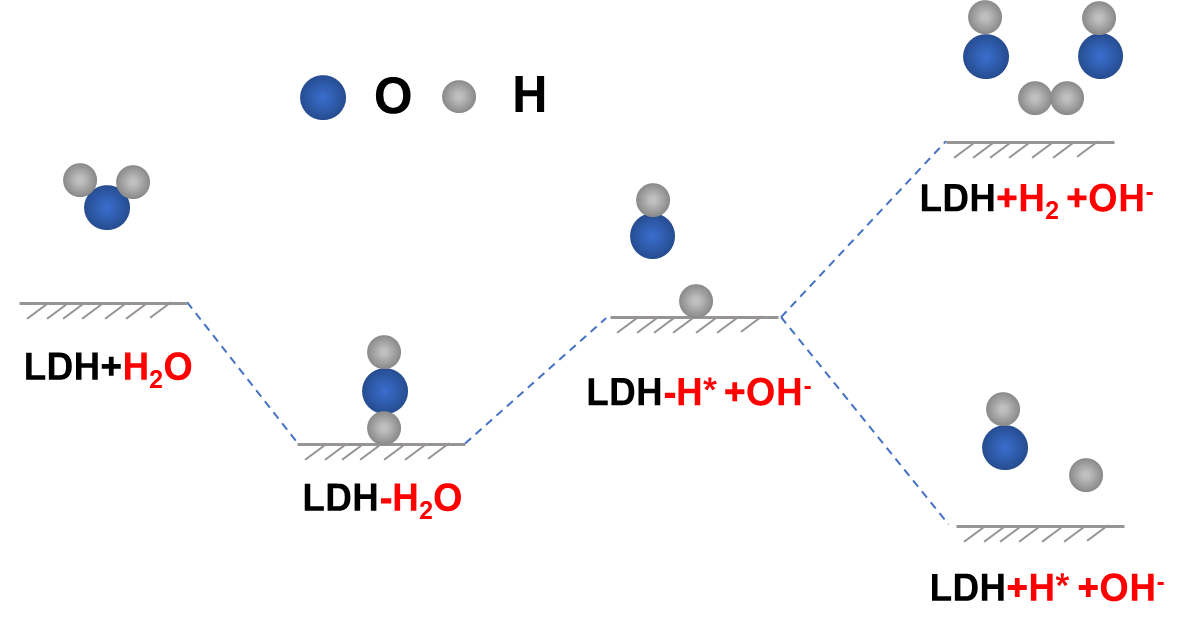


Figure S32 Reactive hydrogen or hydrogen production during the decomposition of H_2_O is schematically depicted.


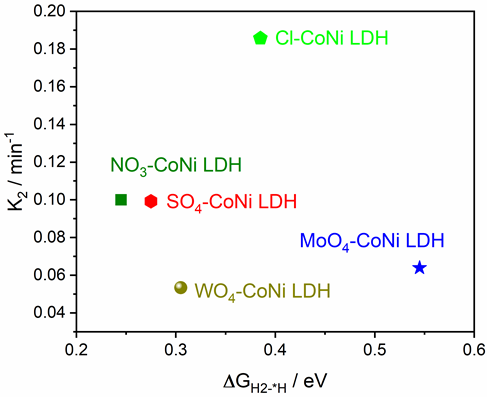


Figure S33 The correlation between the rate constant of nitrite reduction to ammonia (*k*_2_) and hydrogen release form *H was investigated.





Figure S34 Average discharge voltage and corresponding energy density at varying nitrate concentrations.


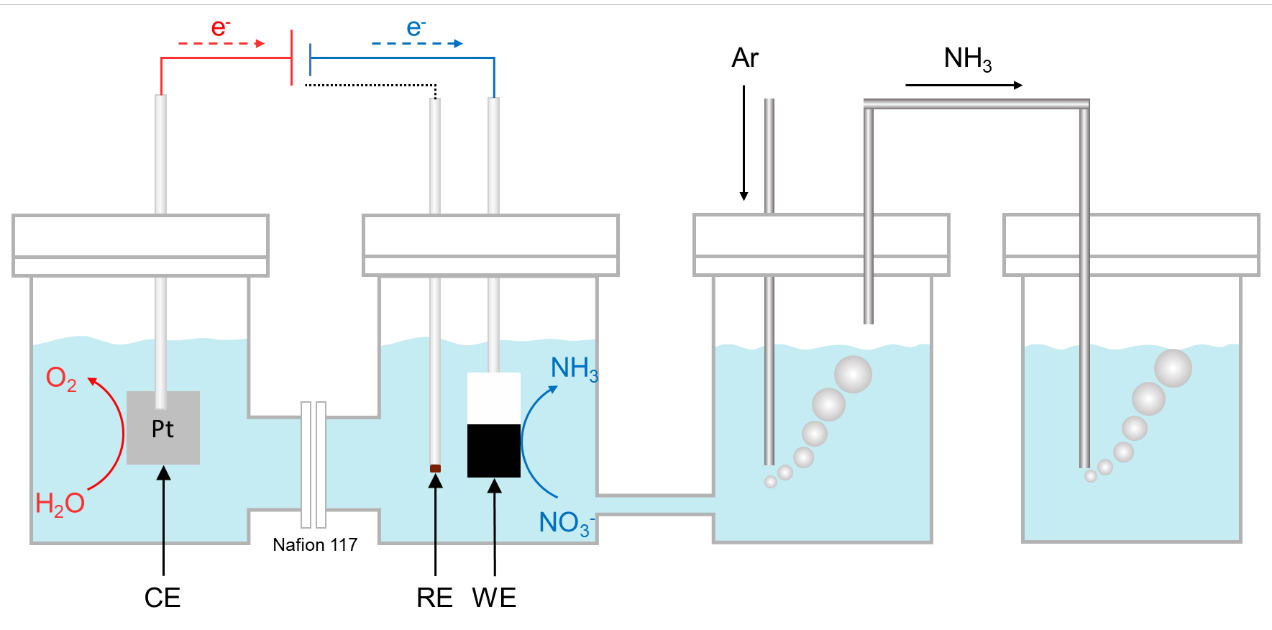


Figure S35 Schematic of the ammonia product synthesis process from nitrate-containing influent to NH_4_Cl(s).

**3 Supporting Tables**

**Table S1** The content ratio (wt%) between Co, Ni and the central element of intercalated anions in NO_3_-NiCo LDH/CuO NW/CF, Cl-NiCo LDH/CuO NW/CF, SO_4_-NiCo LDH/CuO NW/CF, MoO_4_-NiCo LDH/CuO NW/CF and WO_4_-NiCo LDH/CuO NW/CF.

| Electrode | Element | Wt/% |
| --- | --- | --- |
| Cl-CoNi LDH/CuO NW/CF | Cl | 5.65 |
|  | Cu | 15.02 |
|  | O | 21.73 |
|  | Co | 23.29 |
|  | Ni | 25.68 |
| NO_3_-CoNi LDH/CuO NW/CF | N | 4.23 |
|  | Cu | 9.99 |
|  | O | 29.56 |
|  | Co | 28.00 |
|  | Ni | 28.22 |
| SO_4_-CoNi LDH/CuO NW/CF | S | 7.03 |
|  | Cu | 8.61 |
|  | O | 23.10 |
|  | Co | 30.35 |
|  | Ni | 30.91 |
| MoO_4_-CoNi LDH/CuO NW/CF | Mo | 10.06 |
|  | Cu | 10.61 |
|  | O | 24.78 |
|  | Co | 27.53 |
|  | Ni | 27.02 |
| WO_4_-CoNi LDH/CuO NW/CF | W | 10.36 |
|  | Cu | 11.93 |
|  | O | 20.84 |
|  | Co | 28.65 |
|  | Ni | 28.22 |

**Table S2** UV-vis absorption data obtained from tests of different electrode products

| NO_2_^-^ absorbance/a.u. | Multiple of dilution | NH_3_ absorbance/a.u. | Multiple of dilution |
| --- | --- | --- | --- |
| 0.53008 | 50 | 0.16780 | 800 |
| 0.15712 | 50 | 0.16814 | 800 |
| 0.31739 | 52 | 0.16722 | 800 |
| 0.61368 | 100 | 0.16024 | 800 |
| 0.40791 | 100 | 0.16883 | 800 |
| 0.57510 | 200 | 0.16093 | 800 |
| 0.21081 | 1000 | 0.14102 | 800 |
| 0.30502 | 1000 | 0.13862 | 800 |
| 0.28219 | 1000 | 0.13999 | 800 |
| 0.37704 | 2000 | 0.16169 | 400 |
| 0.46996 | 2000 | 0.16379 | 400 |
| 0.42141 | 2000 | 0.16516 | 400 |
| 0.45613 | 2000 | 0.06762 | 200 |
| 0.49793 | 2000 | 0.06441 | 200 |
| 0.47478 | 2000 | 0.06858 | 200 |
| 0.49022 | 500 | 0.09284 | 20 |
| 0.46578 | 500 | 0.08231 | 20 |
| 0.40791 | 500 | 0.09284 | 20 |

**Table S3** Comparison of NitRR performance over reported catalysts.

| Materials | Yield_NH3_/mmol·cm^−2^·h^−1^ | FE _NH3_/% | Reference |
| --- | --- | --- | --- |
| **MoO_4_-CoNi LDH/CuO NW/CF** | **1.12** | **99.78** | **This work** |
| Cu/Cu_2_O NWs | 0.2449 | 95.8 | ^13^ |
| Cu/Cu_2_O | 0.2198 | 93.9 | ^14^ |
| Cu nanosheets | 1.01 | 95 | ^15^ |
| Cu-RD-KOH | 0.1839 | 96.5 | ^16^ |
| Cu_2_O+CO_3_O_4_-CP | 0.9114 | 85.4 | ^17^ |
| Ni_3_Fe-CO_3_ LDH/Cu | 1.261 | 96.8 | ^18^ |
| NiFe-LDH-OV | 0.015 | 65.2 | ^19^ |
| NiCo LDH/Cu NW | 2.35 | 94.25 | ^20^ |
| CoNi LDHs | 0.195 | 94.65 | ^21^ |

**Table S4** EIS parameters of MoO_4_-CoNi LDH/CuO NW/CF

| Potential/V vs. RHE | *R*_s_/Ω | *R*_ct_/Ω | CPE/mF |
| --- | --- | --- | --- |
| OCP | 40392 | 29.63 | 0.30594 |
| 0.2 | 0.61113 | 38.36 | 0.88078 |
| 0.1 | 0.60041 | 3.451 | 0.89739 |
| 0 | 0.588 | 0.94561 | 0.89599 |
| −0.1 | 0.56271 | 1.231 | 0.83881 |
| −0.2 | 0.56704 | 1.151 | 0.86996 |
| −0.3 | 0.56681 | 0.55714 | 0.74332 |

**Table S5** Comparison of Zn-NO_3_ battery performance over reported catalysts.

| Materials | Power density / mW·cm^−2^ | Refeence |
| --- | --- | --- |
| **MoO_4_-CoNi LDH/CuO NW/CF** | **22.7** | **This work** |
| Fe/Ni2P | 3.25 | ^22^ |
| Ir SAC-Co_3_O_4_ | 5.6 | ^23^ |
| Ni1Cu-SAA | 12.7 | ^24^ |
| NiCo_2_O_4_ | 3.94 | ^25^ |
| ISAA | 12.64 | ^26^ |
| CuTABQ | 12.3 | ^27^ |
| DM-Co | 25 | ^28^ |

**4 Supporting References**

1. Guo S*, et al.* Insights into Nitrate Reduction over Indium-Decorated Palladium Nanoparticle Catalysts. *ACS Catal.* **8**, 503-515 (2018).

2. Yao F*, et al.* Highly selective electrochemical nitrate reduction using copper phosphide self-supported copper foam electrode: Performance, mechanism, and application. *Water Res.* **193**, 116881 (2021).

3. Li X*, et al.* Sub-nm RuOx Clusters on Pd Metallene for Synergistically Enhanced Nitrate Electroreduction to Ammonia. *ACS Nano* **17**, 1081-1090 (2023).

4. Wu Z*, et al.* Co-Catalytic Metal-Support Interactions Design on Single-Atom Alloy for Boosted Electro-Reduction of Nitrate to Nitrogen. *Adv. Funct. Mater.* **n/a**, 2406917 (2024).

5. Wang Y*, et al.* Structurally Disordered RuO2 Nanosheets with Rich Oxygen Vacancies for Enhanced Nitrate Electroreduction to Ammonia. *Angew. Chem., Int. Ed.* **61**, e202202604 (2022).

6. Luo H*, et al.* Relay Catalysis of Fe and Co with Multi-Active Sites for Specialized Division of Labor in Electrocatalytic Nitrate Reduction Reaction. *Adv. Funct. Mater.* **n/a**, 2403838 (2024).

7. Cheng X-F*, et al.* Coordination Symmetry Breaking of Single-Atom Catalysts for Robust and Efficient Nitrate Electroreduction to Ammonia. *Adv. Mater.* **34**, 2205767 (2022).

8. Kresse G*, et al.* Efficiency of ab-initio total energy calculations for metals and semiconductors using a plane-wave basis set. *Comput. Mater. Sci.* **6**, 15-50 (1996).

9. Kresse G*, et al.* Efficient iterative schemes for ab initio total-energy calculations using a plane-wave basis set. *Phys. Rev. B* **54**, 11169-11186 (1996).

10. Blöchl PE. Projector augmented-wave method. *Phys. Rev. B* **50**, 17953-17979 (1994).

11. Wu Y*, et al.* Unexpected monoatomic catalytic-host synergetic OER/ORR by graphitic carbon nitride: density functional theory. *Nanoscale* **11**, 5064-5071 (2019).

12. Bajdich M*, et al.* Theoretical Investigation of the Activity of Cobalt Oxides for the Electrochemical Oxidation of Water. *J. Am. Chem. Soc.* **135**, 13521-13530 (2013).

13. Wang Y*, et al.* Unveiling the Activity Origin of a Copper-based Electrocatalyst for Selective Nitrate Reduction to Ammonia. *Angew. Chem., Int. Ed.* **59**, 5350-5354 (2020).

14. Zhou N*, et al.* Potential-Induced Synthesis and Structural Identification of Oxide-Derived Cu Electrocatalysts for Selective Nitrate Reduction to Ammonia. *ACS Catal.* **13**, 7529-7537 (2023).

15. Fu Y*, et al.* Enhancing Electrochemical Nitrate Reduction to Ammonia over Cu Nanosheets via Facet Tandem Catalysis. *Angew. Chem., Int. Ed.* **62**, e202303327 (2023).

16. Jiang H*, et al.* Enabled Efficient Ammonia Synthesis and Energy Supply in a Zinc–nitrate Battery System by Separating Nitrate Reduction Process into Two Stages. *Angew. Chem., Int. Ed.* **62**, e202218717 (2023).

17. Zhang J*, et al.* Single-entity Electrochemistry Unveils Dynamic Transformation during Tandem Catalysis of Cu2O and Co3O4 for Converting NO3− to NH3. *Angew. Chem., Int. Ed.* **135**, e202214830 (2022).

18. Kim K-H*, et al.* Energy-efficient electrochemical ammonia production from dilute nitrate solution. *Energy Environ. Sci.* **16**, 663-672 (2023).

19. Wang K*, et al.* Intentional corrosion-induced reconstruction of defective NiFe layered double hydroxide boosts electrocatalytic nitrate reduction to ammonia. *Nature Water* **1**, 1068-1078 (2023).

20. Zhang X*, et al.* Tandem Nitrate Electroreduction to Ammonia with Industrial-Level Current Density on Hierarchical Cu Nanowires Shelled with NiCo-Layered Double Hydroxide. *ACS Catal.* **13**, 14670-14679 (2023).

21. Li H*, et al.* Modulating the Surface Concentration and Lifetime of Active Hydrogen in Cu-Based Layered Double Hydroxides for Electrocatalytic Nitrate Reduction to Ammonia. *ACS Catal.*, 12042-12050 (2024).

22. Zhang R*, et al.* Efficient Ammonia Electrosynthesis and Energy Conversion through a Zn-Nitrate Battery by Iron Doping Engineered Nickel Phosphide Catalyst. *Adv. Energy Mater.* **12**, 2103872 (2022).

23. Jin T*, et al.* Mechanochemical-tuning size dependence of iridium single atom and nanocluster toward highly selective ammonium production. *Chem Catalysis* **3**, 100477 (2023).

24. Cai J*, et al.* Electrocatalytic nitrate-to-ammonia conversion with ~100% Faradaic efficiency via single-atom alloying. *Appl. Catal. B Environ.* **316**, 121683 (2022).

25. Liu Q*, et al.* Ambient Ammonia Synthesis via Electrochemical Reduction of Nitrate Enabled by NiCo2O4 Nanowire Array. *Small* **18**, 2106961 (2022).

26. Xie M*, et al.* Intermetallic Single-Atom Alloy In–Pd Bimetallene for Neutral Electrosynthesis of Ammonia from Nitrate. *J. Am. Chem. Soc.* **145**, 13957-13967 (2023).

27. Zhang R*, et al.* Molecular Engineering of a Metal-Organic Polymer for Enhanced Electrochemical Nitrate-to-Ammonia Conversion and Zinc Nitrate Batteries. *Angew. Chem., Int. Ed.* **62**, e202309930 (2023).

28. Lin W*, et al.* A High Power Density Zn-Nitrate Electrochemical Cell Based on Theoretically Screened Catalysts. *Adv. Funct. Mater.* **32**, 2209464 (2022).
